# A metabolic complex of acyltransferase enzymes involved in tomato acylsugar biosynthesis

**DOI:** 10.1093/jxb/erag180

**Published:** 2026-04-17

**Authors:** Varun Dwivedi, Ernest Okertchiri, Adam Yokom, Craig A Schenck

**Affiliations:** Department of Biochemistry, University of Missouri, Columbia, MO 65201, USA; Interdisciplinary Plant Group, University of Missouri, Columbia, MO 65201, USA; Department of Biochemistry, University of Missouri, Columbia, MO 65201, USA; Department of Biochemistry, University of Missouri, Columbia, MO 65201, USA; Department of Biochemistry, University of Missouri, Columbia, MO 65201, USA; Interdisciplinary Plant Group, University of Missouri, Columbia, MO 65201, USA; Max Planck Institute for Molecular Plant Physiology, Germany

**Keywords:** Acylsugar acyltransferases, acylsugars, metabolic complex, protein–protein interactions, *Solanum lycopersicum*, specialized metabolites, tomato

## Abstract

Specialized metabolites mediate diverse plant–environment interactions. Recent work has begun to enzymatically characterize entire plant specialized metabolic pathways; however, little is known about how different pathway components organize and interact within the cell. Here we use acylsugars—a class of specialized metabolites—to explore metabolic complex formation. In *Solanum lycopersicum* (tomato), four trichome-localized acylsugar acyltransferases (SlASAT1– SlASAT4) sequentially add acyl chains to a sucrose core leading to accumulation of tri- and tetra-acylated sucroses. Confocal microscopy demonstrates that tomato ASATs localize to distinct subcellular locations, including the mitochondria, cytosol, and endoplasmic reticulum. To explore pairwise protein–protein interactions in acylsugar biosynthesis, we used various techniques relying on different interaction principles, including co-immunoprecipitation, split-luciferase assays, and bimolecular fluorescence complementation, all demonstrating pairwise SlASAT interactions. Following transient expression of SlASAT1–SlASAT4 in *Nicotiana benthamiana*, we were able to pull down a complex consisting of SlASAT1–SlASAT4, which was confirmed through proteomics. Size exclusion chromatography of the SlASAT pulldown suggests a heteromultimeric complex of ∼300 kDa. This study sheds light on the metabolic coordination of acylsugar biosynthesis through formation of a metabolic complex enabling production of chemical defenses.

## Teaser

Acylsugar biosynthesis in tomato depends on SlASAT relocalization and protein interactions to enable efficient production of tri- and tetra-acylsugars.

## Introduction

Plants produce a wide variety of structurally and functionally diverse metabolites. These compounds support plant growth, development, and adaptation to changing conditions, including biotic and abiotic stresses (Fiesel *et al*., 2022). While advances in comparative transcriptomics and mass spectrometry have enabled the identification of entire plant specialized metabolic pathways (Nett *et al*., 2020), the regulatory mechanisms and interactions between enzymes in these pathways are not well understood. One way in which metabolic pathways are regulated is through formation of protein–protein interaction networks. These multienzyme assemblies increase the local accumulation of metabolites to enable faster reaction rates, isolate intermediates from competing pathways, and protect the cell from reactive metabolites (Sweetlove and Fernie, 2018; Zhang and Fernie, 2021). Metabolic complexes have been identified for some metabolic pathways in plants and other organisms (Zhang and Fernie, 2021) including diverse classes of specialized metabolites such as flavonoids, alkaloids, and cyanogenic glycosides (Stavrinides *et al*., 2015; Dastmalchi *et al*., 2016; Laursen *et al*., 2016; Fujino *et al*., 2018). A key feature of some metabolic complexes is scaffolding proteins that help stabilize the complex and allow coordination across subcellular compartments (Boccia *et al*., 2024; Jozwiak *et al*., 2024).

Acylsugars are a class of specialized metabolites that play a crucial role in the intricate interactions between plants and their environment. Their widespread distribution across the economically important *Solanaceae* family provides a key line of defense for important crops such as tomato (Fan *et al*., 2019). The accumulation of acylsugars on glandular trichomes in the aerial parts of plants provides protection against biotic and abiotic stresses (Luu *et al*., 2017; Fan *et al*., 2019; Feng *et al*., 2022; Moghe *et al*., 2023; Vendemiatti *et al*., 2024). Acylsugars have a backbone sugar, typically sucrose or glucose, with acyl groups of varying lengths, esterified at multiple hydroxyl positions (Vendemiatti *et al*., 2025). Acylsugar biosynthesis can be separated into three distinct phases (Vendemiatti *et al*., 2025). (i) The upstream phase generates the acyl-CoA precursors from branched-chain amino acids (Walters and Steffens, 1990; Kroumova and Wagner, 2003). (ii) The core phase esterifies the acyl-CoAs to the sugar core by four trichome-localized acylsugar acyltransferases (SlASAT1–SlASAT4) of the BAHD family. This family derives its name from the first letters of its first four biochemically characterized members, which mainly catalyze and regulate specialized metabolism through acylation (D’Auria, 2006). (iii) The modification phase includes enzymes that can cleave disaccharides and acyl chains (Schilmiller *et al*., 2016; Leong *et al*., 2019). Sequential action of SlASAT1–SlASAT4 leads to the production of mono-, di-, tri-, and tetra-acylated sucrose derivatives *in vitro*; however, only tri- and tetra-acylsugars accumulate *in planta* (Fan *et al*., 2016). Although, trichome-localized ASATs have been characterized in tomato and across the *Solanaceae* (Nadakuduti *et al*., 2017; Schenck *et al*., 2022), little is known about the localization and coordination of the core acylsugar pathway.

Here, we investigate potential metabolic complex formation in tomato trichome-localized acylsugar biosynthesis. Tomato acylsugar biosynthesis provides an excellent model to explore metabolic complex formation because intermediates do not accumulate and the core pathway is enzymatically characterized. Here, we confirm direct SlASAT interactions using complementary protein–protein interaction assays: bimolecular fluorescence complementation (BiFC), split-luciferase complementation, co-immunoprecipitation (Co-IP), size exclusion chromatography (SEC), and proteomics. These findings using diverse interaction techniques that rely on different biochemical principles which were conducted under different physiological conditions both *in vitro* and *in planta* provide support for an acylsugar metabolic complex in tomato consisting of SlASAT1–SlASAT4. The formation of an acylsugar metabolic complex may enable efficient production of important defense metabolites in tomato.

## Materials and methods

### Subcellular localization of SlASATs

To determine the subcellular localization of SlASAT1–SlASAT4, the full-length coding sequence for each gene was cloned using gene-specific primers (Supplementary Table S1) and transiently expressed in *Nicotiana benthamiana* leaves and Arabidopsis protoplasts. Briefly, the inserts were cloned into the pCAM-YFP and pCAM-RFP plasmids using Gibson assembly, and their expression was driven by the CAM35s promoter, with the C-terminal enhanced yellow fluorescent protein (YFP) and red fluorescent protein (RFP) tags. For protoplast preparation (Yoo *et al*., 2007), Arabidopsis plants (ecotype Columbia-0) were grown under 16 h light at 23 °C and 50% relative humidity. Leaves were thinly sliced with a razor blade and incubated with an enzyme solution containing 1% (w/v) cellulase (Goldbio), 0.25% (w/v) macerozyme (Goldbio), 0.4 M mannitol, 20 mM MES (pH 5.7), and 20 mM KCl incubated for 10 min at 55 °C, and then 0.1% (w/v) BSA and 10 mM CaCl_2_ were added. The cut leaf tissues were incubated for 3–4 h at room temperature. The cell digest was filtered through 70 µm nylon mesh, and the protoplasts were harvested by centrifugation for 2 min at 100 *g*, washed twice in 5 ml of ice-cold W5 solution (154 mM NaCl, 125 mM CaCl_2_, 5 mM KCl, 5 mM glucose, and 2 mM MES, pH 5.7), and then incubated with 2 ml of W5 solution for 30 min on ice. The pellet (protoplasts) was suspended in MMG solution (15 mM MgCl_2_, 0.4 M mannitol, and 4 mM MES, pH 5.7). Plasmid DNA (10 μg), either individually or combined with organelle-specific markers (or other SlASATs) in a 1:1 ratio, was added to 100 μl of protoplasts. Subsequently, 110 μl of a polyethylene glycol (PEG) solution containing 40% PEG-4000 (Sigma), 0.4 M mannitol, and 0.1 M CaCl_2_ was gently mixed with the protoplast–DNA mixture and incubated for 10–15 min at room temperature. The transfection reaction was stopped using 440 μl of W5 solution and then pelleted by centrifugation for 2 min at 100 *g*. The pellet was resuspended in 500 μl of WI solution (20 mM KCl, 0.5 M mannitol, and 4 mM MES, pH 5.7) and incubated at 22 °C for 16–20 h (Dwivedi *et al*., 2022). For subcellular localization in *N. benthamiana*, the constructs were transformed in *Agrobacterium tumefaciens* GV3101. A single colony of each construct was used to inoculate a 5 ml aliquot of LB medium culture supplemented with 50 μg ml^–1^ kanamycin, 15 μg ml^–1^ rifampicin, and 50 μg ml^–1^ gentamycin. The bacterial cultures were incubated overnight at 28 °C with shaking at 200 rpm. The cultures were then centrifuged at 3000 *g* for 10 min, and the cell pellets were washed once with 5 ml of infiltration buffer (10 mM MES pH 5.7, 10 mM MgCl_2_, 200 μM acetosyringone) and incubated for 1 h in the dark before being used to infiltrate 3-week-old *N. benthamiana* leaves. For combinatorial infiltrations, the OD_600_ for each strain was set to 0.5. The *N. benthamiana* leaves were co-infiltrated, in a 1:1 ratio, with an organelle marker and the desired construct, and visualized after 72 h. The following organelle-specific markers were used for analysis: mitochondrial marker (cytochrome *c* oxidase IV–RFP), endoplasmic reticulum (ER) marker (AtWAK2–RFP-HDEL), peroxisomal marker (RFP–IHHPRELSRL), and Golgi marker (AtGT14–RFP) (Van Der Krol and Chua, 1991; Nelson *et al*., 2007; Falter *et al*., 2019). Free YFP was used as a cytosolic marker with RFP-tagged SlASATs. Additionally, we used a mitochondrial tracking dye (MitoView™ 405, Biotium) to detect mitochondria. Confocal microscopy of Arabidopsis protoplasts or whole *N. benthamiana* leaves was performed using a Leica TCS SP8 inverted confocal microscope, with a ×100 oil immersion and ×20 dry objective. Fluorescence of each construct was recorded separately, and the images were merged to determine co-localization between marker protein and SlASATs. The excitation wavelength and emission bandwidth recorded for each fluorescent protein as well as chlorophyll autofluorescence were optimized by the default pre-sets in the Leica 2.6 software (Leica) and were as follows: eYFP (excitation 514 nm, emission 525–575 nm), RFP (excitation 558 nm, emission 570–620 nm), and chlorophyll autofluorescence (excitation 633 nm, emission 650–725 nm). For co-transfection experiments involving multiple SlASATs, we co-expressed either SlASAT1–YFP or SlASAT3–YFP with SlASAT2 (no fluorescent tag) in a 1:1 molar ratio, alongside an organelle-specific marker, utilizing PEG-mediated transformation in Arabidopsis protoplasts. To generate SlASAT1 lacking the mitochondrial targeting signal, we predicted the signal peptide using WoLF PSORT and TargetP (Supplementary Fig. S1). We then cloned the truncated SlASAT1 into the pCAM plasmid via Gibson assembly. All experiments were repeated three times independently, and the results were similar.

### Bimolecular fluorescence complementation assay

SlASATs were cloned into the pCAM-nYFP and pCAM-cYFP plasmids, which incorporate the N-terminal (amino acids 1–174) and C-terminal (amino acids 175–239) segments of eYFP, respectively. Specifically, SlASAT1 and SlASAT3 were cloned into the pCAM-nYFP construct, both upstream and downstream of nYFP. Similarly, SlASAT2 and SlASAT4 were cloned into the pCAM-cYFP construct, both upstream and downstream of cYFP. *Nicotiana benthamiana* leaves were then co-infiltrated with a 1:1:1 combination of *A. tumefaciens* harboring the ER RFP marker, pCAM-nYFP, and pCAM-cYFP constructs as described above and in the Results. YFP fluorescence was monitored 72 h post-infiltration using confocal microscopy, as done for the subcellular localization studies. Interactions were evaluated by testing all possible combinations in both directions. To identify false interactions, we included the mitochondrial-localized *Atropa belladonna* BAHD acyltransferase (OP677554.1; AbTS) and tomato enoyl-CoA hydratase (SOLYC07G043680.3.1; SlAECH1). For interaction with cytosolic-localized proteins, we included tomato GLYCOALKALOID METABOLISM 36 (SOLYC08G075210.1; SlGAME36) as a negative control. Additionally, each construct was assayed with an empty vector control to monitor any background fluorescence resulting from interactions between the protein of interest and YFP fragments. The BiFC assay was performed using three biological replicates, with leaf infiltration done on separate plants.

### Split-luciferase complementation imaging assay

The plasmid pCAM-nLUC/cLUC was digested using *Nco*I to clone SlASAT1 and SlASAT3 downstream of pCAM-nLUC, and with *Swa*I to clone SlASAT2 and SlASAT4 upstream of pCAM-cLUC. Similar to BiFC, we included AbTS, SlAECH1, and SlGAME36 as negative controls. The generated constructs were sequenced using Sanger sequencing to verify the correct sequence. The nLUC and cLUC constructs harboring the respective genes of interest were transformed into *A. tumefaciens* (GV3101) and infiltrated into *N. benthamiana* as detailed above. For combinatorial infiltrations, the OD_600_ for each strain was set to 0.5. After 48–72 h, the infiltrated leaves were used for the LUC activity measurement. Coelenterazine (0.25 mM; Goldbio) was sprayed once onto the leaves, and they were kept in the dark for 7 min to allow chlorophyll luminescence to decay. The luminescence was then monitored using a Photek 216 (Photek, Ltd). All experiments were independently repeated three times, with leaf infiltrations performed on separate plants.

### Pulldown protein complexes putatively formed *in vivo*

To perform the *in vivo* pulldown of SlASAT1–SlASAT4, the full-length coding sequences for each target were amplified by PCR from tomato genomic DNA using hemagglutinin (HA)- and FLAG-incorporated primers listed in Supplementary Table S1. To check for negative interactions, we incorporated FLAG tag on AbTS and SlAECH1, and an HA tag on SlGAME36. The DNA fragments were amplified using Phusion DNA Polymerase. PCR products were purified using a PCR cleanup kit (Qiagen) and cloned into the pCAM plasmid. The constructs were then transformed into *A. tumefaciens* (GV3101) competent cells. The transformants were grown on LB plates containing 50 μg ml^–1^ kanamycin, 15 μg ml^–1^ rifampicin, and 50 μg ml^–1^ gentamycin at 28 °C. A single colony was used to inoculate 5 ml of LB medium supplemented with antibiotics and grown overnight at 28 °C. The cells were centrifuged at 3000 *g* for 10 min. The pellet was resuspended in 5 ml of infiltration medium (10 mM MES pH 5.7, 10 mM MgCl_2_, 200 μM acetosyringone), incubated at room temperature for 2 h, and subsequently used to infiltrate 3-week-old leaves. For combinatorial infiltrations, the OD_600_ for each strain was set to 0.5. The leaves were harvested 3 d post-infiltration, frozen in liquid nitrogen, and stored at −80 °C. For the detection of pairwise SlASAT interactions, proteins were extracted by homogenizing leaf tissue in a buffer containing 150 mM Tris–HCl (pH 7.5), 150 mM NaCl, 5 mM EDTA, 1 mM phenylmethylsulfonyl fluoride (PMSF), and 1% Nonidet P-40 (NP-40), along with a protease inhibitor cocktail (Halt™, Thermo Fisher Scientific). The homogenates were then clarified by centrifugation at 16 000 *g* for 25 min at 4 °C, and the soluble proteins were incubated with anti-HA (Pierce™ Anti-HA Magnetic Beads) or anti-FLAG (Pierce™ Anti-DYKDDDDK Magnetic Agarose) magnetic beads for either 1 h at room temperature or overnight at 4 °C, following the manufacturer’s protocol. After removing the unbound proteins, the beads were washed three times with a buffer containing 10 mM Tris–HCl (pH 7.5), 150 mM NaCl, 0.5 mM EDTA, and 0.1% SDS. The proteins were recovered by resuspending the beads in 2× Laemmli sample buffer [125 mM Tris–HCl (pH 6.8), 20.0% glycerol, and 4.0% SDS], denaturing them at 100 °C for 5 min. The proteins were separated by 12% SDS–PAGE and detected by immunoblotting with monoclonal mouse anti-FLAG (Sigma) antibodies (1:1500 dilution) or monoclonal mouse anti-HA (Invitrogen) antibodies (1:1500 dilution) and goat anti-mouse IgG horseradish peroxidase (HRP) conjugate (Invitrogen, 1:10 000 dilution) as a secondary antibody. Protein bands were visualized with the Immobilon Western Chemiluminescent HRP Substrate kit (Millipore) and imaged using the Invitrogen™ iBright™ Imaging Systems. After immunodetection, Ponceau S staining was used as a control for equal protein loading. Blotted membranes were incubated in Ponceau S staining solution (Thermo Fisher Scientific) for 5 min, before excess solution was washed away with distilled H_2_O. All experiments were performed in three independent replicates, with leaf infiltrations conducted on separate plants.

### Proteomic analysis of the pulldown complex

The protein complexes from the pulldown were precipitated with 4 volumes of cold acetone and kept overnight at −20 °C. The protein pellets were then washed twice with acetone. Proteins were denatured/reduced with 6 M urea, 2 M thiourea, and 5 mM DTT. Proteins were alkylated with 15 mM iodoacetamide (IAA). Protein was digested with LyC at 1:50 (enzyme:protein ratio) for 3 h at 37 °C and then digested with trypsin (1:50 enzyme:protein ratio) overnight at 37 °C. Digested peptides were purified by Evosep tips. Data were acquired on a Bruker timsTOF Pro2 connected to the Evosep-One system, and data were searched against the *N. benthamiana* protein database concatenated with SlASAT1–SlASAT4 proteins using Spectronaut version 19.3. For all proteome analyses, we used an EvoSep One LC system (Bache *et al*., 2018) and analyzed the samples with a 44 min gradient eluting the peptides (or the Evosep One 30 SPD program). We used a 15 cm×150 μm internal diameter column with 1.5 μm C18 beads (Bruker PepSep) and a 10 µm internal diameter zero dead volume electrospray emitter (Bruker Daltonik). Mobile phases A and B were 0.1% formic acid in water and 0.1% formic acid in acetonitrile, respectively. Evosep One was coupled online to a modified trapped ion mobility spectrometry quadrupole time-of-flight mass spectrometer (timsTOF Pro 2, Bruker Daltonik GmbH, Germany) via a nanoelectrospray ion source (Captive spray, Bruker Daltonik GmbH). To conduct data-independent acquisition-parallel accumulation serial fragmentation (DIA-PASEF), the timsTOF Pro2 operated in long gradient DIA-PASEF mode. Sixteen scans with narrow 25 *m/z* isolation windows (resulting in 32 windows) covered an *m/z* range of 400–1200 and an ion mobility range of 0.6–1.43 V s cm^−2^. The duty cycle was maintained at 100%. This setup results in a total cycle time of 1.8 s (1× 100 ms MS1 survey scan, 16× 100 ms DIA-PASEF scans). All experiments were repeated four times independently, and the results were similar.

### Analysis of the proteomics data

The DIA-PASEF raw data were analyzed using the directDIA workflow of Spectronaut version 19.3 with default settings: trypsin digestion with two allowed missed cleavages, cysteine carbamidomethylation as a fixed modification, methionine oxidation, and acetylation at the protein N-terminus as variable modifications. All searches were conducted against the Uniprot *N. benthamiana* database concatenated with SlASAT1–SlASAT4. Proteins and peptides were filtered at a Q-value of 0.01. Functional enrichment analysis was performed using the QuickGO annotation tool (https://www.ebi.ac.uk/QuickGO/annotations) to classify the proteins into three categories: biological processes, cellular compartments, and molecular functions. For any identified proteins that were not annotated in the UniProt database, the InterProScan software was utilized to annotate their Gene Ontology (GO) functions.

### Size exclusion chromatography of ASAT1–ASAT4 complex

Following expression of SlASAT1–SlASAT4 in *N. benthamiana*, the protein complex was pulled down using FLAG-tagged SlASAT4 or HA-tagged SlASAT1 as bait. Since the proteins were analyzed as complexes, elution was conducted using non-denaturing conditions with FLAG or HA peptide. Samples were subsequently separated under native conditions by SEC using a Superdex 200 3.2/300 column (Cytiva) and equilibrated with 50 mM Tris–HCl, 150 mM NaCl, pH 7.4 at a flow rate of 0.050 ml min^–1^. Prior to loading, samples were concentrated using 30 kDa molecular weight cut-off centrifugal filter units (Millipore, Amicon Ultra Centrifugal Filter). A 50 µl aliquot of the sample was injected onto the column and elution was monitored by UV absorbance at 280 nm; fractions were collected at 100 µl intervals. Molecular mass determination was achieved by comparison with the gel filtration standard protein mixture (BioRad) monitored at UV absorbance of 215 nm under the same elution conditions. All experiments were independently repeated three times with similar results.

## Results

### Tomato ASATs localize to distinct subcellular locations

To experimentally determine the subcellular localization of tomato ASATs, we generated SlASAT fusion proteins with YFP and transiently expressed them in Arabidopsis protoplasts. Prior to experimental determination of SlASAT1–SlASAT4 subcellular localization, we predicted likely locations using subcellular prediction tools to guide placement of YFP (Supplementary Fig. S1). *In silico* analyses provided an indication of subcellular localization, but the results were inconsistent for SlASATs across various prediction tools (Supplementary Fig. S1). The prediction guided placement of YFP, which was fused to the C-terminus of full-length SlASAT coding sequences to minimize the chance of blocking a targeting signal (primers found in Supplementary Table S1).

**Fig. 1.**
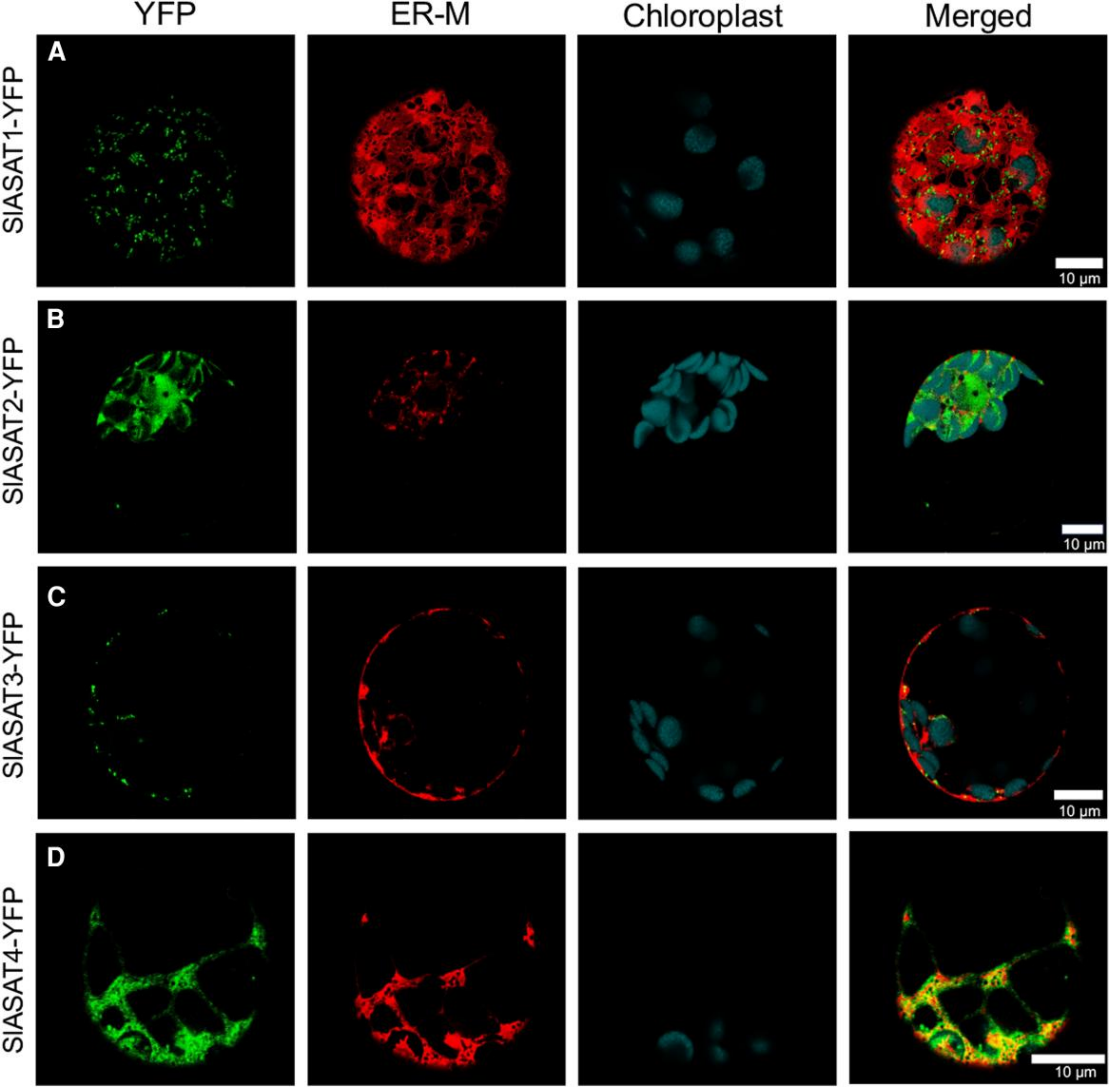
Distributed subcellular localization of SlASAT1–SlASAT4 fusion proteins in Arabidopsis protoplasts. (A) SlASAT1, (B) SlASAT2, (C) SlASAT3, and (D) SlASAT4 fusion constructs (shown on the left) were expressed in Arabidopsis protoplasts and visualized by confocal microscopy. The ‘YFP’ panels represent signals of SlASAT-fused fluorescence proteins; the ‘ER-M’ panels represent the signals of the ER marker (AtWAK2–RFP-HDEL); the ‘chloroplast’ panels represent chlorophyll autofluorescence; and the ‘Merged’ panel shows the overlay of YFP, ER-M, and chloroplast signals. The experiments were repeated three times with similar results, and representative images are shown. A scale bar is shown in each panel of images. Additional imaging with RFP- and YFP-tagged SlASATs and other organelle markers can be found in Supplementary Figs S2–S4.

Previous reports have shown that *Nicotiana tabacum* ASAT1 is localized to the ER (Chang *et al*., 2022). Thus, the localization of YFP-tagged SlASATs was first tested with an ER marker in Arabidopsis protoplasts, providing insight into the nature of the distribution of SlASATs across different organelles (Fig. 1). SlASAT1 and SlASAT3 exhibited a punctate pattern, which did not overlap with an ER marker protein or chlorophyll autofluorescence (Fig. 1). SlASAT2 and SlASAT4 displayed a more continuous and distributed localization pattern, with SlASAT4 co-localizing with the ER marker protein while SlASAT2 did not (Fig. 1). Because of the punctate signal observed for SlASAT1 and SlASAT3 (Fig. 1), we next tested co-localization of SlASAT1 and SlASAT3 with a mitochondrial marker (Supplementary Fig. S2A). Both SlASAT1 and SlASAT3 overlapped with the mitochondrial marker (Supplementary Fig. S2A). To support the mitochondrial localization of SlASAT1, we tested the localization of a truncated version of SlASAT1 without the predicted mitochondrial transit peptide (Supplementary Fig. S2B, C). The truncated version of SlASAT1 displayed a diffuse distribution, in contrast to the punctate pattern of the full-length sequence, which did not overlap with an ER marker (Supplementary Fig. S2B). To further support mitochondrial localization of SlASAT1 and SlASAT3 and not other organelles, we tested their localization with golgi and peroxisome markers, and observed no overlap (Supplementary Fig. S3). To test the localization of SlASATs to the cytosol, we tagged the proteins with RFP and infiltrated the fusion proteins into *N. benthamiana* together with free YFP as a cytosolic marker (Supplementary Fig. S4). Consistent with Arabidopsis protoplast experiments, SlASAT1 and SlASAT3 showed a punctate distribution with some diffuse signal, whereas SlASAT2 and SlASAT4 showed a diffuse distribution signal (Supplementary Fig. S4). Most of the signal from SlASAT1 and SlASAT3 did not overlap with the cytosolic marker, however some cells showed overlap (Supplementary Fig. S4). Together with the localization in protoplasts (Supplementary Fig. S2) that show mitochondrial localization, it is likely that SlASAT1 and SlASAT3 are also localized to the cytosol; however, the primary location appears to be the mitochondria. SlASAT2 showed a diffuse signal that overlapped with the cytosolic marker, demonstrating a cytosolic localization (Supplementary Fig. S4). SlASAT4 signal mostly did not overlap with the cytosolic marker, further demonstrating ER localization (Supplementary Fig. S4). In summary, SlASATs are distributed across various subcellular locations, with SlASAT1 and SlASAT3 primarily localized to the mitochondria, SlASAT2 localized to the cytosol, and SlASAT4 localized to the ER.

### Co-transfection of SlASAT pairs alters subcellular localization

To determine the effect of binary protein–protein interactions on localization patterns, we transfected Arabidopsis protoplasts with combinations of SlASAT pairs, with one partner tagged with YFP and the other untagged. Tagged plasmids expressing SlASAT1 or SlASAT3 were co-transfected with an untagged version of SlASAT2 into Arabidopsis protoplasts. Confocal microscopy revealed that co-expression of SlASAT1 with SlASAT2 resulted in an altered localization of SlASAT1 (Supplementary Fig. S5). We found that SlASAT1 now completely localized to the cytosol, instead of the mitochondria when expressed alone (Supplementary Fig. S5), suggesting that the presence of SlASAT2 altered the localization of SlASAT1 (Supplementary Fig. S5). We also tested the localization of tagged SlASAT3 in combination with SlASAT2, as SlASAT3 was also localized to the mitochondria. Here, we observed a similar change in localization for SlASAT3, which completely localized to the cytosol (Supplementary Fig. S5). These results suggest a potential role for SlASAT2 in protein–protein interactions with other SlASATs, leading to the altered localization of SlASAT1 and SlASAT3. This relocalization phenomenon has been observed for other proteins including aquaporins that relocalize following co-transfection, demonstrating the impacts of protein–protein interactions on altering subcellular localization (Zelazny *et al*., 2007).

### Complementary interaction techniques demonstrate SlASAT pairwise interactions

To test whether pairwise protein–protein interactions occur between all SlASATs, we performed split-luciferase complementation assays by expressing fusion protein pairs in *N. benthamiana* leaves. SlASAT1 and SlASAT3 were fused to the N-terminus of the luciferase fragment, while the potential interacting partners SlASAT2 and SlASAT4 were fused to the C-terminus (Supplementary Fig. S6). The assay indicated interactions between SlASAT1 and SlASAT2, SlASAT2 and SlASAT3, SlASAT3 and SlASAT4, and SlASAT1 and SlASAT4 (Supplementary Fig. S6A). To identify potential false interactions involving SlASATs and other proteins, we included mitochondrially localized proteins from *Atropa belladonna* and tomato. A BAHD acyltransferase from *A. belladonna*, 3β-tigloyloxytropane synthase (AbTS), which is involved in tropane alkaloid biosynthesis (Zeng *et al*., 2024), was included as a negative control as it localizes to the same compartment and comes from the same protein family. Tomato acylsugar enoyl-CoA hydratase (SlAECH1) is involved in producing medium-chain acyl-CoA substrates used by ASATs in acylsugar biosynthesis, and was chosen as a negative control because it is involved in acylsugar biosynthesis and localized to the mitochondria (Fan *et al*., 2020). Both proteins were fused to the C-terminus of the luciferase fragment and showed no interaction with SlASAT1 and SlASAT3 (Supplementary Fig. S6A). Similarly, to check for false interactions with SlASAT2 and SlASAT4, we used tomato GLYCOALKALOID METABOLISM 36 (SlGAME36) as a negative control. SlGAME36, a BAHD acyltransferase which catalyzes acetylation and is involved in glycoalkaloid metabolism (Sonawane *et al*., 2023), showed no interaction with SlASAT2 and SlASAT4 (Supplementary Fig. S6A). Furthermore, we employed an empty vector as an additional negative control and observed no interaction, suggesting that the observed SlASAT interactions are specific (Supplementary Fig. S6A). We further quantified the luciferase assay using protein extracted from *N. benthamiana* infiltrated leaves and observed similar interactions among the SlASATs. Based on the intensity of the relative luciferase units, the strongest interactions were observed between SlASAT1 and SlASAT2, and between SlASAT3 and SlASAT4 (Supplementary Fig. S6B, C).

**Fig. 2.**
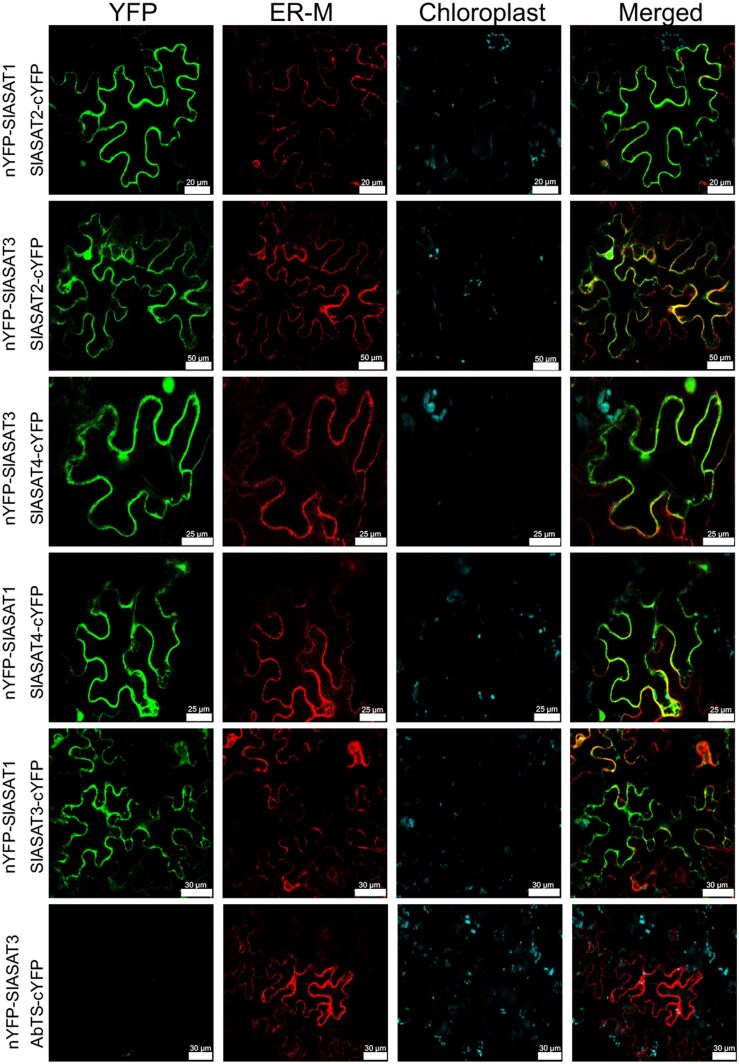
SlASATs show pairwise protein–protein interactions. Bimolecular fluorescence complementation (BiFC) assays were performed with transiently expressed SlASAT pairs in *N. benthamiana.* SlASAT1 and SlASAT3 were fused to N-terminal YFP, while SlASAT2, SlASAT4, and AbTS (negative control) were fused to C-terminal YFP. The ‘YFP’ panels represent signals of SlASAT complexes; the ‘ER-M’ panels represent the signals of the ER marker (AtWAK2–RFP-HDEL); the ‘chloroplast’ panels represent chlorophyll autofluorescence; and the ‘Merged’ panel shows the overlay of YFP, ER-M, and chloroplast signals. A scale bar is shown in the image. The experiments were repeated three times with similar results. Representative images are shown with a scale bar in each image. Additional BiFC images with different locations of tags and additional negative controls can be found in Supplementary Figs S7 and S8.

To further investigate pairwise SlASAT interactions, BiFC assays were performed. Protein interactions were visualized using confocal microscopy, with the presence of fluorescence indicating either proximity or interaction between the enzymes, as well as their subcellular localization. SlASAT1 and SlASAT3 were fused to the C-terminus of nYFP (nYFP–SlASAT1 and nYFP–SlASAT3), while their interacting partners, SlASAT2 and SlASAT4, were fused to the N-terminus of cYFP (SlASAT2–cYFP and SlASAT4–cYFP). Similarly, mitochondrial-localized negative controls, AbTS and SlAECH1, were fused to the N-terminus of the cYFP fragment (AbTS–cYFP and SlAECH1–cYFP), while the SlGAME36 was fused to the C-terminus of nYFP (nYFP–SlGAME36). To test SlASAT1–SlASAT3 interaction, SlASAT3 was additionally fused to the N-terminus of cYFP (SlASAT3–cYFP). The assay showed strong interactions between the tested pairs of SlASAT1 and SlASAT2, SlASAT2 and SlASAT3, SlASAT3 and SlASAT4, SlASAT1 and SlASAT4, and SlASAT1 and SlASAT3 (Fig. 2). No interactions were observed in negative controls (Fig. 2; Supplementary Fig. S7). To check the effect of tag orientation on the interaction between the SlASATs, we moved the position of the tags on the SlASATs so that they were both located at the N-terminus. We found that altering the tag orientation reduced the intensity of the observed interactions, but pairwise interactions were observed for SlASAT1 and SlASAT2, SlASAT2 and SlASAT3, and SlASAT3 and SlASAT4 (Supplementary Fig. S8). The signal for interacting SlASATs localized to the cytosol as signal did not overlap with an ER marker protein, suggesting that pairwise SlASAT interactions are occurring in the cytosol (Fig. 2; Supplementary Fig. S8), consistent with SlASAT1 and SlASAT3 interacting with SlASAT2 in the cytosol (Supplementary Fig. S5). Consistent with split-luciferase assays, strong interactions were detected between the SlASAT1/SlASAT2, SlASAT2/SlASAT3, and SlASAT3/SlASAT4 protein pairs, suggesting that these enzymes may directly interact with each other (Fig. 2). Our results demonstrate that acylsugar pathway enzymes can interact with each other in a pairwise manner.

### Pairwise interactions of SlASATs via co-immunoprecipitation

To examine the interactions among the different SlASATs and further verify pairwise interactions, we performed Co-IP of tagged SlASATs transiently expressed in *N. benthamiana*. We first optimized the expression of the four SlASAT enzymes by individually expressing them in *N. benthamiana* and analyzing their expression levels in the total protein extracts and the immunoprecipitated fractions (Supplementary Figs S9, S10). We confirmed the western blot results through proteomics, which detected 55–86% sequence coverage of the SlASATs in the respective pulldowns (Supplementary Figs S9, S10).

**Fig. 3.**
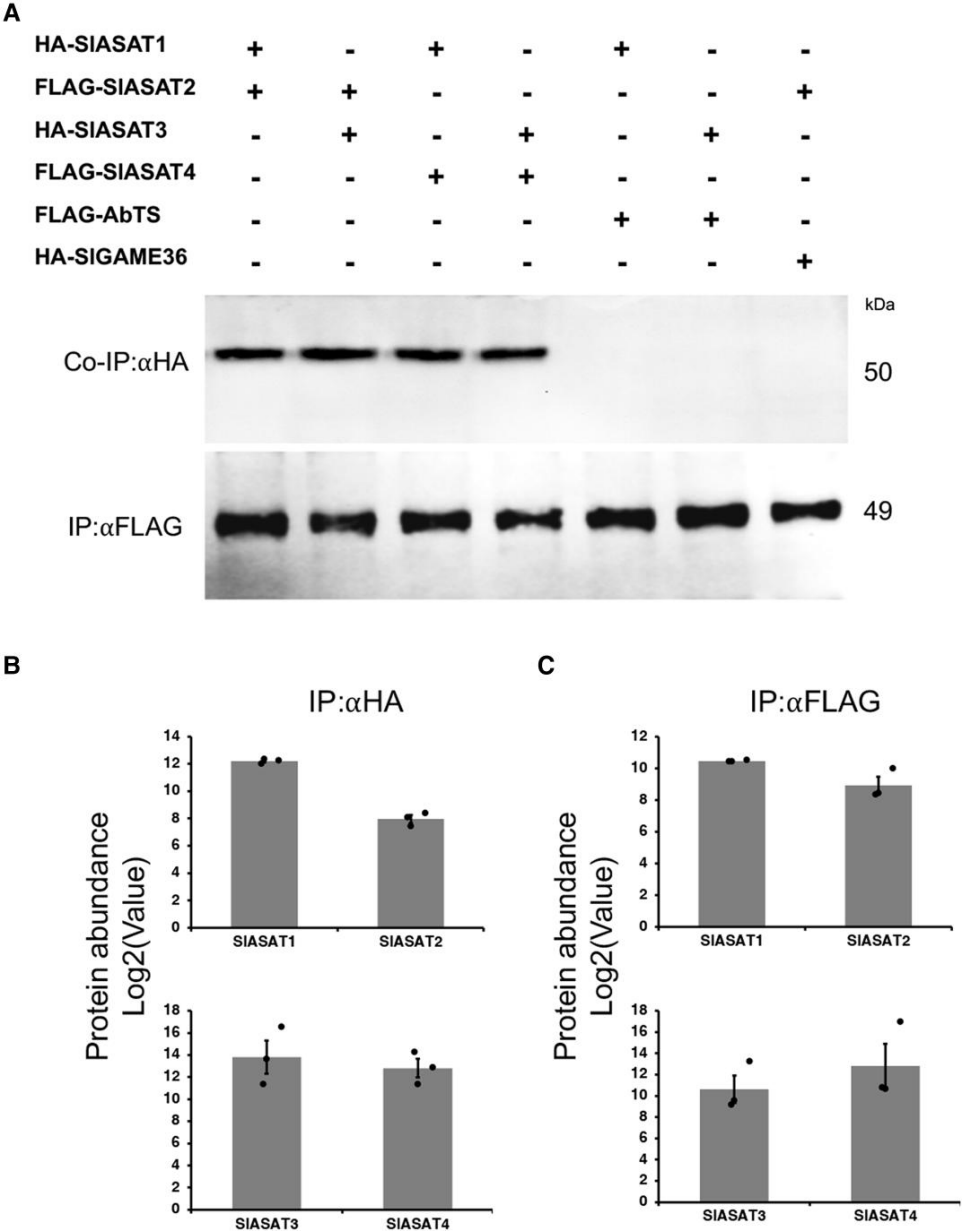
Co-immunoprecipitation of SlASATs confirms pairwise interactions. HA- and FLAG-tagged fusion proteins were transiently expressed in *N. benthamiana* leaves, and total proteins were extracted 3 d after infiltration. Protein extracts were transiently expressed in a pairwise manner: HA-SlASAT1 and FLAG-SlASAT2; HA-SlASAT3 and FLAG-SlASAT2; HA-SlASAT1 and FLAG-SlASAT4; HA-SlASAT3 and FLAG-SlASAT4; HA-SlASAT1 and FLAG-AbTS; HA-SlASAT3 and FLAG-AbTS; and HA-SlGAME36 and FLAG-SlASAT2. (A) Protein gel blot analysis of FLAG-IP samples. Immunoprecipitation (IP) was performed with anti-FLAG magnetic bead conjugate, and interacting proteins were analyzed with an anti-HA antibody. Interactions between SlASATs and negative controls were not observed. (B, C) Quantification of the abundance of the HA/FLAG pull-down metabolic complex using proteomics. Protein quantification was based on peptide spectral counts. Bars indicate the average ±SE of three biological replicates, with individual replicates plotted.

Following optimization, we performed Co-IP experiments to investigate the interactions between the SlASATs. In these experiments, SlASAT1 and SlASAT3 were tagged with HA, and SlASAT2 and SlASAT4 were tagged with FLAG to enable pairwise interaction tests. Additionally, HA-SlGAME36, FLAG-AbTS, and FLAG-SlAECH1 were used as negative controls. When SlASAT1 and SlASAT2 were co-infiltrated in *N. benthamiana* and pulldown was conducted with anti-FLAG magnetic beads, we observed signal using both anti-HA and anti-FLAG antibodies, indicating interaction between the pair (Fig. 3A). Similar interactions were observed between SlASAT2 and SlASAT3, SlASAT1 and SlASAT4, and SlASAT3 and SlASAT4 (Fig. 3A). Interactions between SlASATs and negative control proteins were not observed (Fig. 3A). Pulldowns were also evaluated via proteomics to confirm our results. Proteomics confirmed the Co-IP results detecting both SlASATs in each pairwise interaction (Fig. 3B, C).

To further confirm SlASAT pairwise interactions, we performed Co-IP in a reciprocal manner with anti-HA magnetic beads and observed interactions between SlASAT1 and SlASAT2, and between SlASAT3 and SlASAT4 (Supplementary Fig. S11A). Similarly, SlASAT pairwise interactions were observed when Co-IP with anti-FLAG beads was performed (Supplementary Fig. S11B). The results further demonstrate pairwise interactions between SlASAT1 and SlASAT2, and SlASAT3 and SlASAT4 (Supplementary Fig. S11).

### SlASAT1–SlASAT4 complex formation

**Fig. 4.**
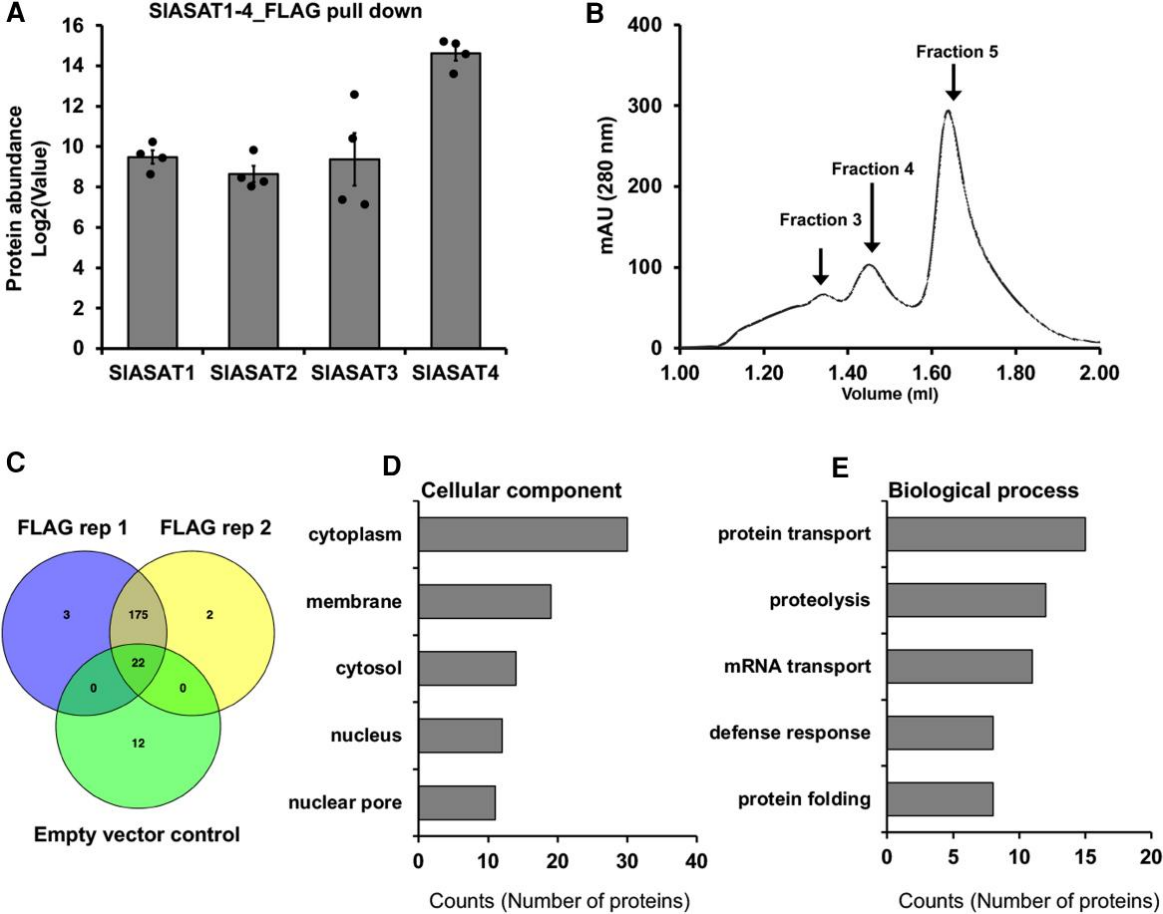
A multimeric SlASAT metabolic complex. HA-tagged SlASAT1 and FLAG-tagged SlASAT4 were infiltrated in *N. benthamiana* together with untagged SlASAT2 and SlASAT3. Protein extracts were prepared from *N. benthamiana* leaves transiently expressing all four SlASATs and immunoprecipitation was performed using either anti-HA or anti-FLAG magnetic bead conjugates. (A) Quantification of the abundance of the FLAG pull-down metabolic complex using proteomics analysis. Protein quantification was based on peptide spectral counts. Bars indicate the average ±SE of four biological replicates, with individual replicates plotted. (B) Size exclusion chromatography (SEC) of the SlASAT1–SlASAT4 complex. The SlASAT pulldown complex was eluted using non-denaturing conditions and separated using SEC. Peaks corresponding to ∼271 kDa (Fraction 3), ∼108 kDa (Fraction 4), and ∼49 kDa (Fraction 5) were identified and used for western blot analysis for both HA and FLAG antibodies (Supplementary Fig. S12). The molecular mass of the fraction was estimated based on protein standards run using an identical method and column (Supplementary Fig. S12). (C) Overlap of lists of interacting candidate proteins identified by LC-MS/MS analysis in two replicates and a negative (empty vector) control. (D and E) Functional enrichment analysis using QuickGo tool (https://www.ebi.ac.uk/QuickGO/annotations) of proteins identified in the SlASAT1–SlASAT4 metabolic complex pulldown experiment.

Given the observed interactions between pairs of SlASATs, we next investigated if all four SlASATs could interact, forming a metabolic complex. We expressed constructs with untagged SlASAT2 and SlASAT3, along with HA-tagged SlASAT1 and FLAG-tagged SlASAT4, in *N. benthamiana* and subsequently performed immunoprecipitation experiments using either HA or FLAG magnetic beads (Fig. 4). The pulldown samples were initially confirmed by western blot and then subjected to proteomic analysis. Our results showed that all four SlASATs were detected in the proteomics data of the pulldowns, indicating the formation of a multi-enzyme complex involved in acylsugar biosynthesis (Fig. 4A). In the FLAG pulldown, SlASAT4 had the highest abundance compared with SlASAT1, SlASAT2, and SlASAT3 (Fig. 4A). The differences in protein abundances observed may indicate the stoichiometry of the SlASAT complex, their expression levels in *N. benthamiana*, or their detectability in our proteomics analysis. These data indicate an acylsugar interaction network consisting of at least SlASAT1–SlASAT4, representing four sequential steps of acylsugar biosynthesis. To gain insights into the size and stoichiometry of the SlASAT protein complex, we performed SEC on the immunoprecipitated SlASAT complex eluted under non-denaturing conditions. Molecular mass standards for globular proteins were run prior to the SlASAT samples (Supplementary Fig. S12). As shown in Fig. 4B, we observed three main protein peaks. These peaks correspond to a molecular mass of 271, 108, and 49 kDa, respectively (Fig. 4B). These data suggest a multimeric assembly consisting of SlASATs (the approximate molecular mass of each SlASAT is 50 kDa). Western blot of the peak fractions with HA and FLAG antibodies (targeting SlASAT1 and SlASAT4, respectively) demonstrate that SlASAT1 and SlASAT4 are found in the peak corresponding to the largest molecular mass, as well as other fractions (Supplementary Fig. S12). Together, the SEC and western blot analyses suggest a heteromultimeric SlASAT complex (Fig. 4; Supplementary Fig. S12).

### Identification of additional interacting proteins in the SlASAT1–SlASAT4 complex

Although our aim with Co-IP followed by proteomics was to characterize the SlASAT1– SlASAT4 metabolic complex, we could also use this experimental setup to identify additional potential interacting proteins. To identify proteins interacting with the SlASAT complex, we conducted a proteomics analysis of all proteins co-immunoprecipitated with the pulldown of SlASAT4 (Fig. 4C). Four biological replicates were prepared, with pulldown protein extracted using high detergent stringency for the SlASAT replicates and the empty vector controls. By mapping all detected proteins to the *N. benthamiana* proteome, we identified 175 unique proteins in the pulldown complex that were putatively interacting with SlASATs that were not present in the empty vector HA/FLAG negative control (Fig. 4C). To identify potential protein interactions, we hypothesized that those with abundant proteins detected in the pulldown are more likely to be true interactors. The relative abundances of the potential candidate proteins identified in the pull-down experiments are shown in Supplementary Fig. S13 and Supplementary Table S2. The most abundant proteins detected in the pulldowns were involved in fatty acid biosynthesis, including acetyl-CoA C-acetyltransferase and acyl-[acyl-carrier-protein] desaturase. Additionally, we identified transporter proteins such as ABC-type transporters and NbRanBP1-1a, which may play a role in shuttling intermediates in acylsugar biosynthesis. Furthermore, we identified proteins involved in mitochondria-associated ER membrane contact sites. These proteins represent potential interactors with the SlASAT acylsugar complex. To understand if the pulldown of the SlASAT metabolic complex was enriched in proteins with other functions, we performed a functional enrichment analysis of the overlapping proteins in our pulldown replicates. Functional enrichment analysis for cellular process revealed enrichment of cytoplasm/cytosol and membrane, among other terms (Fig. 4D). Functional enrichment for biological process indicated an enrichment of proteins related to transport (Fig. 4E), implying the diverse localization of the different complex components and potential interacting transporters in the ASAT complex.

## Discussion

Since plants cannot rapidly evade harsh conditions, they rely on specialized metabolites to adapt and thrive in diverse environments. Multi-enzyme complexes coordinating structurally diverse plant specialized metabolites have been characterized, for example in flavonoid biosynthesis (Dastmalchi *et al*., 2016; Mameda *et al*., 2018), phenylproponaoid biosynthesis (Achnine *et al*., 2004), monoterpene indole alkaloids (Stavrinides *et al*., 2015), cyanogenic glycosides (e.g. dhurrin) (Laursen *et al*., 2016), terpenoids (Camagna *et al*., 2019), and steroidal glycoalkaloids (Boccia *et al*., 2024). Here, we identified a multi-enzyme complex in tomato responsible for acylsugar biosynthesis, thereby adding another group of metabolites coordinated via metabolic complex formation. Tomato contains four trichome-enriched BAHD acyltransferases, SlASAT1–SlASAT4, which sequentially acylate sucrose to produce tetra-acylsucroses (Schilmiller *et al*., 2012, 2015; Fan *et al*., 2016). Despite the structural simplicity of acylsugar precursors, the *Solanaceae* family exhibits remarkable acylsugar diversity (Fan *et al*., 2019; Moghe *et al*., 2023). This diversity is attributed to variations in the acylation pattern, branching pattern, and chain length (2–16 carbons) of the acyl groups. The SlASAT1–SlASAT4 enzymes produce sequential acylation at the R4 (monoacylated), R3 (diacylated), R3′ (triacylated), and R2 (tetra-acylated) positions, respectively, to generate acylated sucrose molecules (Fan *et al*., 2016, 2019; Schenck and Last, 2020). The acyl-CoA substrates for each SlASAT are distributed across different organelles.

The upstream pathways that provide the acyl chains for ASATs are localized to various subcellular compartments (Kroumova and Wagner, 2003; Vendemiatti *et al*., 2025). The branched-chain amino acids, which are the precursors to acyl chains, are biosynthesized in the plastids (Walters and Steffens, 1990; Kroumova and Wagner, 2003). Prior to esterification, the branched-chain amino acids are converted into their subsequent acyl-CoAs by the branched-chain α-keto acid dehydrogenase complex in the mitochondria (Mooney *et al*., 2002). These small CoAs are then esterified onto the sugar backbone by ASATs (Schilmiller *et al*., 2012, 2015; Fan *et al*., 2016). BAHD enzymes involved in flavonoid biosynthesis are known to localize to the cytosol (Dhaubhadel *et al*., 2008; Yu *et al*., 2008; Bontpart *et al*., 2015). In soybean, GmMT7, an isoflavonoid malonyltransferase catalyzing malonylation, localizes to the cytosol (Dhaubhadel *et al*., 2008). Similarly, in *Medicago truncatula*, MtMaT1, catalyzing the malonylation of flavonoid glucosides, was localized to both the cytoplasm and the nucleus (Yu *et al*., 2008). Recently, in *A. belladonna*, a BAHD acyltransferase, 3β-tigloyloxytropane synthase, which catalyzes the formation of 3β-tigloyloxytropane from 3β-tropanol and tigloyl-CoA, a key intermediate in calystegine biosynthesis, was shown to be localized to the mitochondria (Zeng *et al*., 2024). Similarly, Chang *et al*. (2022) characterized ASAT1 and ASAT2 from *N. tabacum*, which are localized in the ER (Chang *et al*., 2022). The subcellular localization of BAHD acyltransferases seems to be as divergent as their enzymatic activities (Bontpart *et al*., 2015).

In this study, we demonstrate that SlASAT1 and SlASAT3 primarily localize to the mitochondria when expressed alone, whereas SlASAT2 and SlASAT4 localize to the cytosol and ER (Fig. 1; Supplementary Figs S2, S3, S4). However, upon interaction, SlASAT1 and SlASAT3 predominantly relocalize to the cytoplasm (Fig. 2; Supplementary Fig. S5). Protein relocalization upon protein interaction has been documented previously, for instance, aquaporins and sucrose transporters alter their subcellular localization upon co-expression and interaction (Zelazny *et al*., 2007; Krügel *et al*., 2008; Krügel and Kühn, 2013). Likewise, in *Arabidopsis thaliana*, the initial enzyme of the shikimate pathway, 3-deoxy-D-arabino-heptulosonate 7-phosphate synthase, (DAHPS), relocalizes to the cytosol upon interacting with partner proteins (Kanaris *et al*., 2022). We used various complementary experimental approaches, including BiFC, split-luciferase assays, and Co-IP, to confirm pairwise protein–protein interactions among SlASATs (Figs 2, 3; Supplementary Figs S6, S11). Additionally, we were able to pull down the entire SlASAT complex and further validated these findings through proteomics and SEC analysis (Fig. 4; Supplementary Fig. S12). These results confirm the formation of a metabolic complex involving SlASATs. This close association is essential for the acylsugar biosynthetic pathway to produce biologically active acylsugars with their specific esterified acyl-CoA. However, signal was observed in SEC fractions with approximate monomeric molecular masses (Fig. 4). Thus, we have observed an intact complex, but also individual components, suggesting an equilibrium between association and disassociation of the acylsugar metabolic complex. The tight association of SlASATs could confer several advantages, such as sequestering of pathway intermediates and substrates (acyl-CoAs) to minimize competition from other pathways and thereby substantially enhance the efficiency of acylsugar biosynthesis. We cannot rule out the possibility of a metabolon in acylsugar biosynthesis and potential substrate channeling in this pathway, but this requires further validation. Additionally, a recent study showed that expression of SlASAT1–SlASAT3 in *Escherichia coli* enabled acylsugar production (Han *et al*., 2025). We hypothesize that the close association of these proteins is essential for enhancing acylsugar production with specific acyl-CoAs and reducing precursor/intermediate diffusion.

Metabolic complexes consist of the core metabolic enzymes, but often contain other proteins that are required for complex assembly (Zhang and Fernie, 2021). In addition to SlASATs representing the core portion of acylsugar biosynthesis, other proteins were observed in pulldowns; some probably represent true contributors to the SlASAT metabolic complex whereas others are likely to be contaminants. The potential candidate proteins were annotated to be involved in transport, fatty acid biosynthesis, and specialized metabolism (Supplementary Fig. S13; Supplementary Table S2). Our functional enrichment analysis revealed an abundance of proteins involved in biological processes such as protein transport (Fig. 4E), which could be responsible for the interorganellar transport of pathway intermediates and acyl-CoA substrates required for acylsugar biosynthesis. Similarly, we observed an enrichment of proteins associated with cytosolic and membrane-related functions (Fig. 4D). These proteins may directly or indirectly affect the metabolic complex (Fig. 4). Further experiments are needed to follow-up on additional proteins that may be involved in the identified acylsugar metabolic complex.

**Fig. 5.**
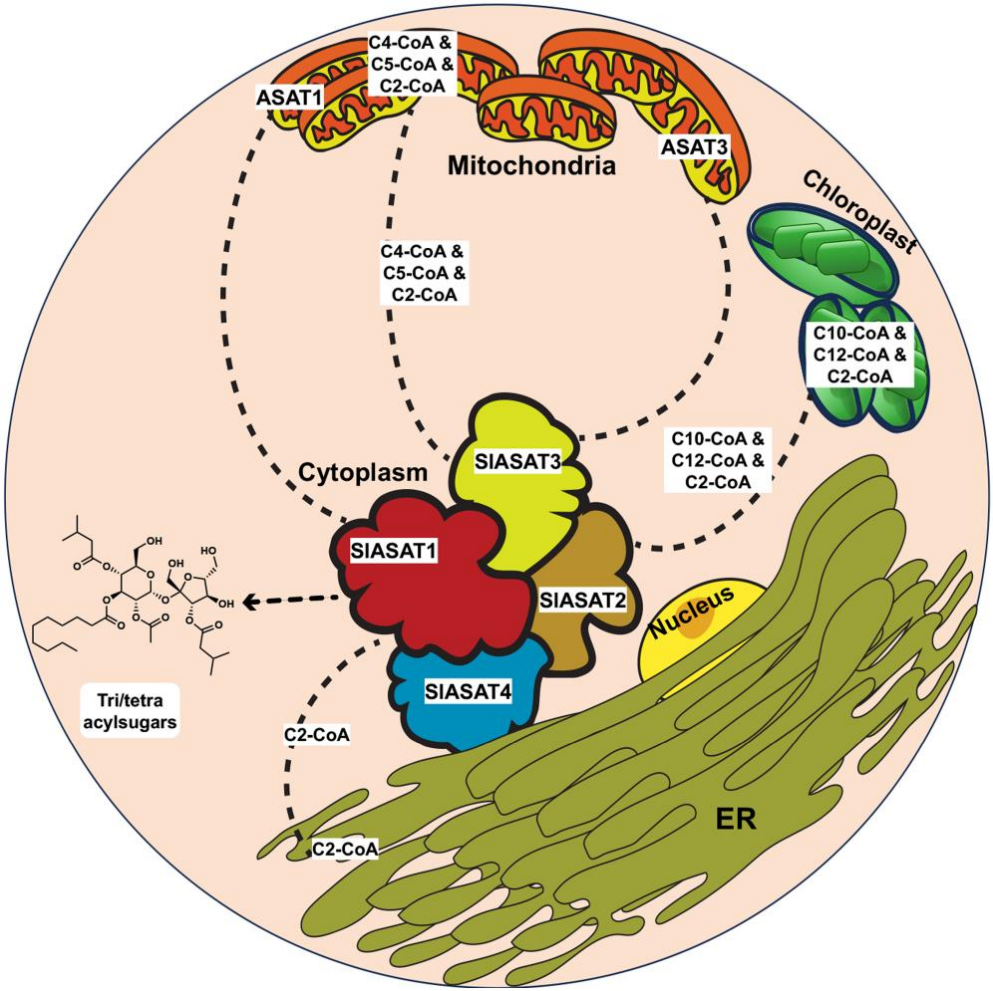
Proposed model of SlASAT metabolic complex formation. SlASAT1 and SlASAT3 localize primarily to mitochondria, whereas SlASAT2 and SlASAT4 localize to the cytosol and ER, respectively. Dashed lines represent the flow of acyl-CoA substrates. SlASAT2, localized to the cytosol, may act as a key hub that links other SlASATs localized to different compartments and re-localize SlASAT1 and SlASAT3 to the cytosol. A black dashed line with an arrow indicates the release of tri- and tetra-acylsugars from the ASAT metabolic complex.

We have demonstrated a metabolic complex for tomato trichome-localized acylsugar biosynthesis (Fig. 5). An ASAT complex may be conserved across the *Solanaceae* species that make acylsugars. However, each species has its own acylsugar biosynthetic differences, for example the presence of additional enzymes, rearrangement of enzymatic order, use of different acyl chains and sugar cores, localization to different organs, and many others that remain unknown (Fan *et al*., 2017, 2019; Schenck *et al*., 2022; Nowack *et al*., 2026). Thus, if an ASAT metabolic complex is conserved across the *Solanaceae*, it may appear slightly different in a species-specific manner. Structural modeling and high-throughput proteomics approaches could be used to validate ASAT complex formation in other species.

## Supplementary Material

erag180_Supplementary_Data

## Data Availability

The MS proteomics data have been deposited to the ProteomeXchange Consortium via the PRIDE partner repository with the dataset identifier PXD072183.

## Supplementary data

The following supplementary data are available at *JXB* online.

Fig. S1. *In silico* subcellular localization prediction of tomato ASATs using different tools.

Fig. S2. SlASAT1 and SlASAT3 fusion proteins in Arabidopsis protoplasts are localized to the mitochondria.

Fig. S3. Subcellular localization of SlASAT1 and SlASAT3 fusion proteins in Arabidopsis protoplasts with other organelle markers.

Fig. S4. Subcellular localization of SlASAT1–SlASAT4 RFP-tagged proteins in *N. benthamiana* leaves with a cytosolic marker.

Fig. S5. SlASAT2 alters SlASAT1 and SlASAT3 subcellular localization to the cytosol.

Fig. S6. SlASAT protein–protein interactions via split-luciferase assays in *N. benthamiana*.

Fig. S7. SlASAT protein–protein interactions via BiFC in *N. benthamiana* with an ER marker.

Fig. S8. Moving fluorescent tag locations demonstrates SlASAT pairwise interaction via BiFC assays in *N. benthamiana.*

Fig. S9. SlASAT1 and SlASAT2 pulldown optimization.

Fig. S10. SlASAT3 and SlASAT4 pulldown optimization.

Fig. S11. Confirmation of pairwise SlASAT interactions via Co-IP.

Fig. S12. Size exclusion chromatography of the SlASAT pulldown complex.

Fig. S13. List of candidate proteins identified through proteomics analysis of the SlASAT1–SlASAT4 metabolic complex.

Table S1. Primers used in the study.

Table S2. Proteomics result of the SlASAT pulldown complex with SlASATs highlighted.
