# Supplementary material for: A metabolic complex of acyltransferase enzymes involved in tomato acylsugar biosynthesis
Source: J Exp Bot. 2026 Apr 17;77(14):4620–33. doi: 10.1093/jxb/erag180 (PMC13415957; doi:10.1093/jxb/erag180)
Supplement: erag180_Supplementary_Data [file erag180_supplementary_data.zip › JXB_Supp_proof_NEW.pdf]

Supplementary Information For:

A metabolic complex of acyltransferase enzymes involved in tomato acylsugar biosynthesis

Authors: Varun Dwivedi[1,2], Ernest Okertchiri[1], Adam Yokom[1], Craig A. Schenck[1,2,3]

[1]Department of Biochemistry, University of Missouri, Columbia, MO, USA

[2]Interdisciplinary Plant Group, University of Missouri, Columbia, MO, USA

[3]Corresponding author, caschenck@missouri.edu

Target P	Other	Signal peptide	Mitochondrial transfer peptide	Chloroplast transfer peptide	Thylakoid luminal transfer peptide
**SlASAT1**	0.2079	0.0003	0.772	0.0181	0.0016
**SlASAT2**	0.4022	0.0018	0.2518	0.2355	0.1086
**SlASAT3**	0.5482	0.0083	0.4001	0.0377	0.0058
**SlASAT4**	0.9833	0.0166	0.0001	0	0

Deeploc-2.0	Cytoplasm	Nucleus	Extracellular	Cell membrane	Mitochondrion	Plastid	ER	Lysosome/Vacuole	Golgi apparatus	Peroxisome
**SlASAT1**	0.4505	0.5484	0.0551	0.1241	0.0688	0.2130	0.2970	0.0625	0.0286	0.0415
**SlASAT2**	0.4617	0.4944	0.0436	0.1514	0.0842	0.2555	0.2426	0.0929	0.0241	0.0319
**SlASAT3**	0.4091	0.5563	0.0535	0.1971	0.1003	0.2341	0.2529	0.0782	0.0188	0.0533
**SlASAT4**	0.4041	0.4905	0.0660	0.2431	0.0685	0.2103	0.3693	0.0986	0.0616	0.1052

WoLF PSORT	Chlo	Mito	Chlo_mito	Cyto	Cyto_mito	Nuc	Cysk	Pero
**SlASAT1**	chlo: 6	mito: 5.5	-	-	cyto_mito: 3.5	nucl: 2	-	-
**SlASAT2**	chlo: 8.5	mito: 3	chlo_mito: 6.33333	cyto: 1	cyto_mito: 2.33333	nucl: 1	-	-
**SlASAT3**	chlo: 2.5	mito: 6	chlo_mito: 4.83333	cyto: 1	cyto_mito: 3.83333	nucl: 3	cysk: 1	-
**SlASAT4**	chlo: 2	mito: 1	-	cyto: 9	-	nucl: 1	-	pero: 1

Predotar	Mitochondrial	Plastid	ER	Elsewhere	Prediction
**SlASAT1**	0,70	0,01	0,01	0,30	mitochondrial
**SlASAT2**	0,31	0,02	0,00	0,68	possibly mitochondrial
**SlASAT3**	0,26	0,01	0,01	0,73	possibly mitochondrial
**SlASAT4**	0,05	0,00	0,00	0,94	none

Plant-mPLoc	SlASAT1	SlASAT2	SlASAT3	SlASAT4
**SlASATs**	Cytoplasm	Cytoplasm	Cytoplasm	Cytoplasm

Fig. S1. *In silico* subcellular localization prediction of tomato ASATs using different tools. The subcellular localization was predicted using several tools, with the results indicated by the organelle scores. The scores represent the reliability of the prediction. **TargetP** (https://services.healthtech.dtu.dk/services/TargetP-2.0/); **Deeploc-2.0** (https://services.healthtech.dtu.dk/services/DeepLoc-2.0/); **WoLF PSORT** (https://wolfpsort.hgc.jp/); **Predotar** (https://urgi.versailles.inra.fr/predotar/); **Plant-mPLoc** (http://www.csbio.sjtu.edu.cn/bioinf/plant-multi/).

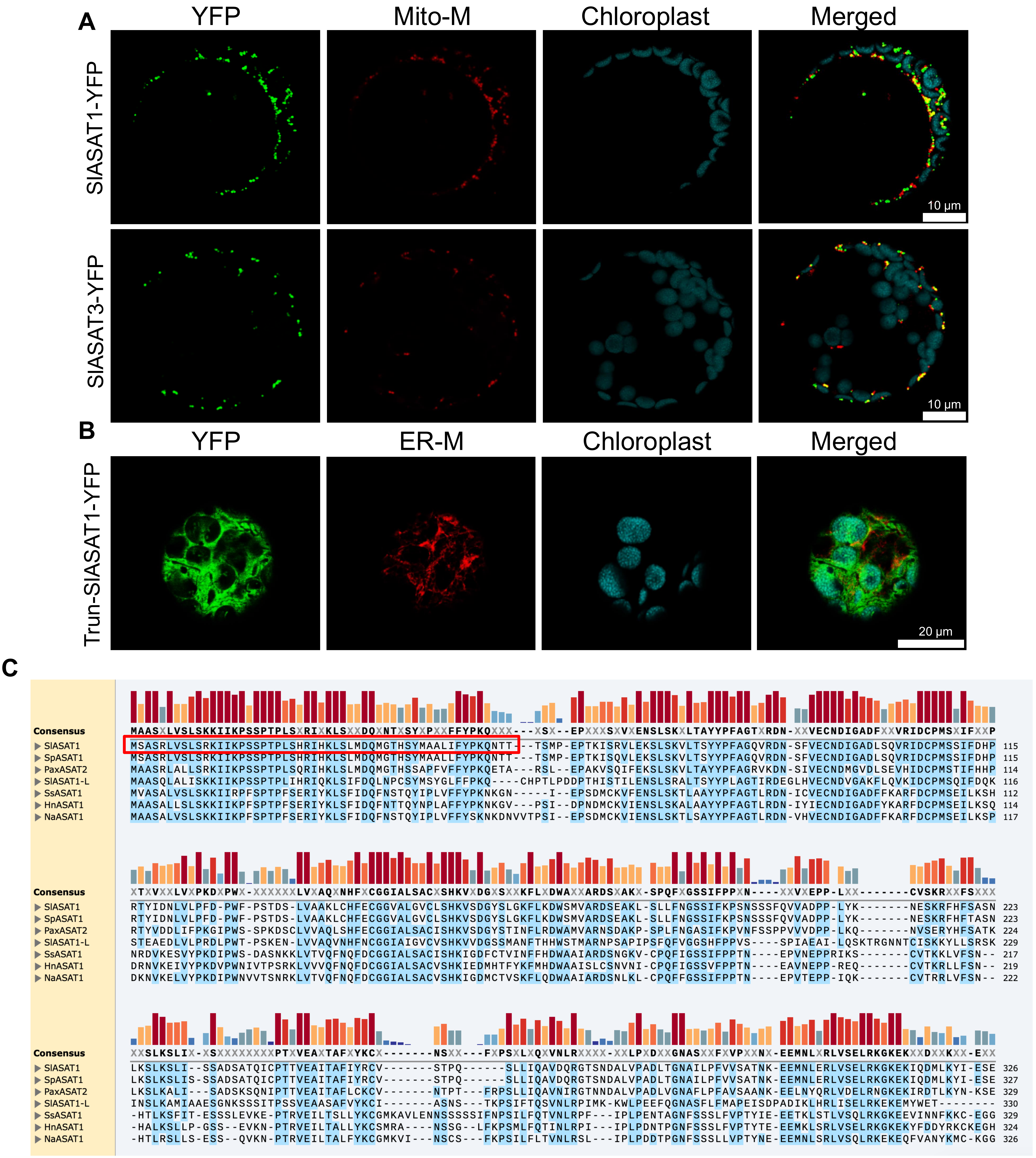

A
YFP
Mito-M
Chloroplast
Merged
SlASAT1-YFP
SlASAT3-YFP
10 μm
10 μm
B
YFP
ER-M
Chloroplast
Merged
Trun-SlASAT1-YFP
20 μm
C
Consensus
SlASAT1
SpASAT1
PaxASAT2
SlASAT1-L
SsASAT1
HnASAT1
NaASAT1

Fig. S2. SlASAT1 and SlASAT3 fusion proteins in Arabidopsis protoplasts are localized to the mitochondria. **A)** SlASAT1 and SlASAT3 fusion proteins (shown on the left) were expressed in Arabidopsis protoplasts, and the localization of the corresponding protein was detected by confocal laser scanning microscopy. **B)** SlASAT1-YFP without mitochondrial peptide fusion constructs (shown on the left, "Trun-SlASAT1-YFP"). The "YFP" panels (green) represent signals of SlASATs fused fluorescence proteins; the "Mito-M" panels (red) represent the signals of mitochondrial marker (MitoView™ Dye); the "ER-M" panels (red) represent the signals of ER marker (AtWAK2- RFP-HDEL); the "chloroplast" panels (cyan) represent chlorophyll autofluorescence. The "Merged" panel shows the overlay of YFP, organelle marker, and chloroplast signals. The experiments were repeated three times with similar results and representative images are shown. A scale bar is shown in each panel of images. **C)** Amino acid multiple sequence alignment of SlASAT1 homologs across different Solanaceae species. The sequence of SlASAT1 shows the predicted mitochondrial targeting signal highlighted in a red box. SlASAT1; *Solanum lycopersicum*, SpASAT1; *Solanum pennellii*, SlASAT1-L; *Solanum lycopersicum*-Like, SsASAT1; *Salpiglossis sinuata*, HnASAT1; *Hyoscyamus niger*, PaASAT2; *Petunia axillaris* , NaASAT1; *Nicotiana attenuata*. The bars above the alignment represent sequence identity, with red indicating 100%, orange (60-75%), yellow (50-60%), light blue (25-50%), and dark blue (< 20%).

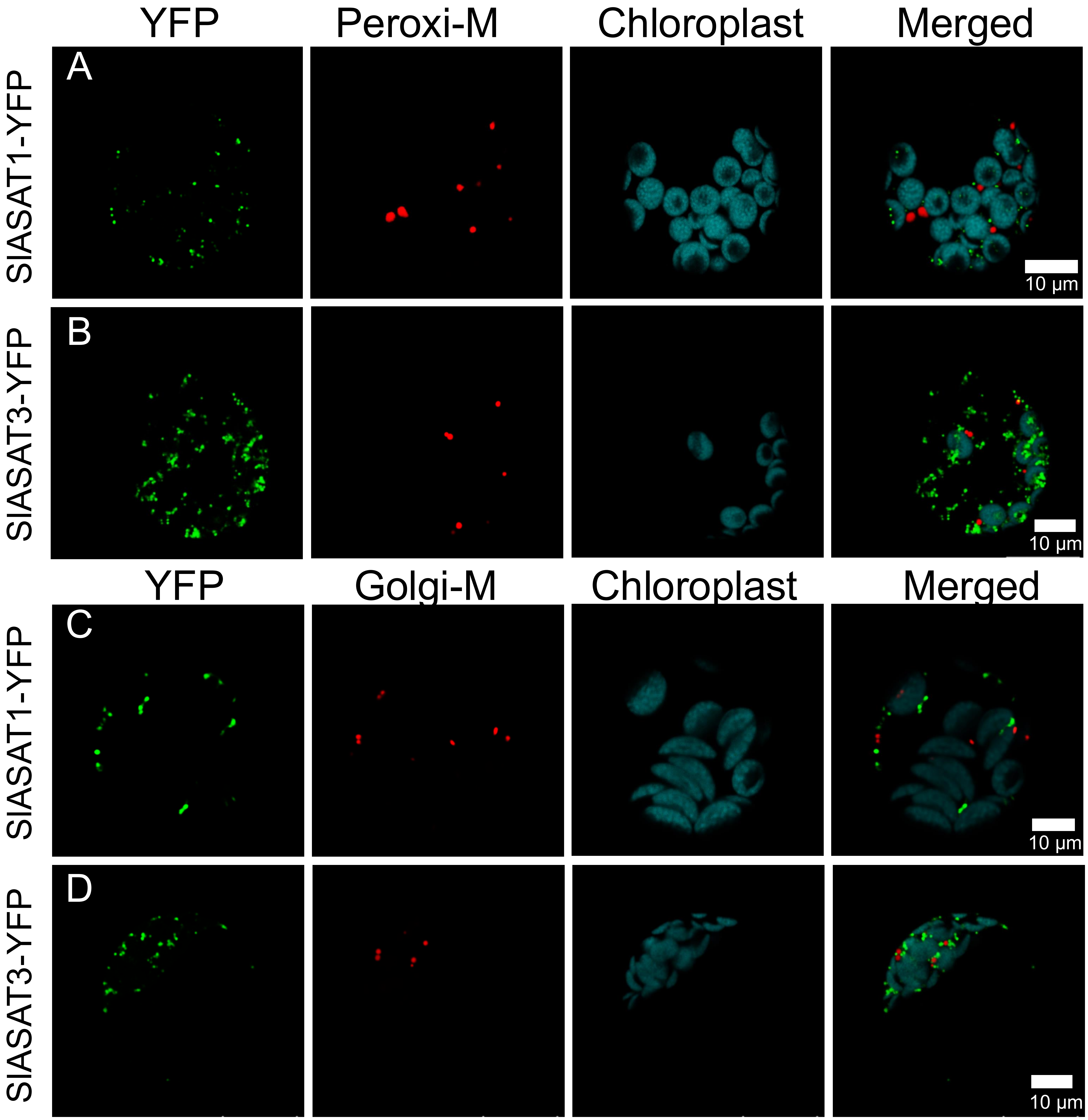

Fig. S3. Subcellular localization of SlASAT1 and SlASAT3 fusion proteins in Arabidopsis protoplasts with other organelle markers. The SlASAT1-YFP and SlASAT3-YFP fusion constructs (shown on the left) were expressed in Arabidopsis protoplasts, and the localization of corresponding proteins was detected by confocal laser scanning microscopy. Panels **A-B)** depict co-expression of SlASAT1 and SlASAT3 with a peroxisome marker, while panels **C-D)** show co-expression of SlASAT1 and SlASAT3 with a golgi marker. The "YFP" panels (green) represent signals of SlASAT1 and SlASAT3 fused fluorescence proteins; the "Peroxi-M" panels (red) represent the signals of peroxisome marker (RFP-IHHPRELSRL); the "Golgi-M" panels (red) represent the signals of golgi marker (AtGT14-RFP); the "chloroplast" panels (cyan) represent chlorophyll autofluorescence signal. The "Merged" panel shows the overlay of YFP, organelle marker, and chloroplast signals. Representative images are shown with a scale bar shown in each panel of images.

.

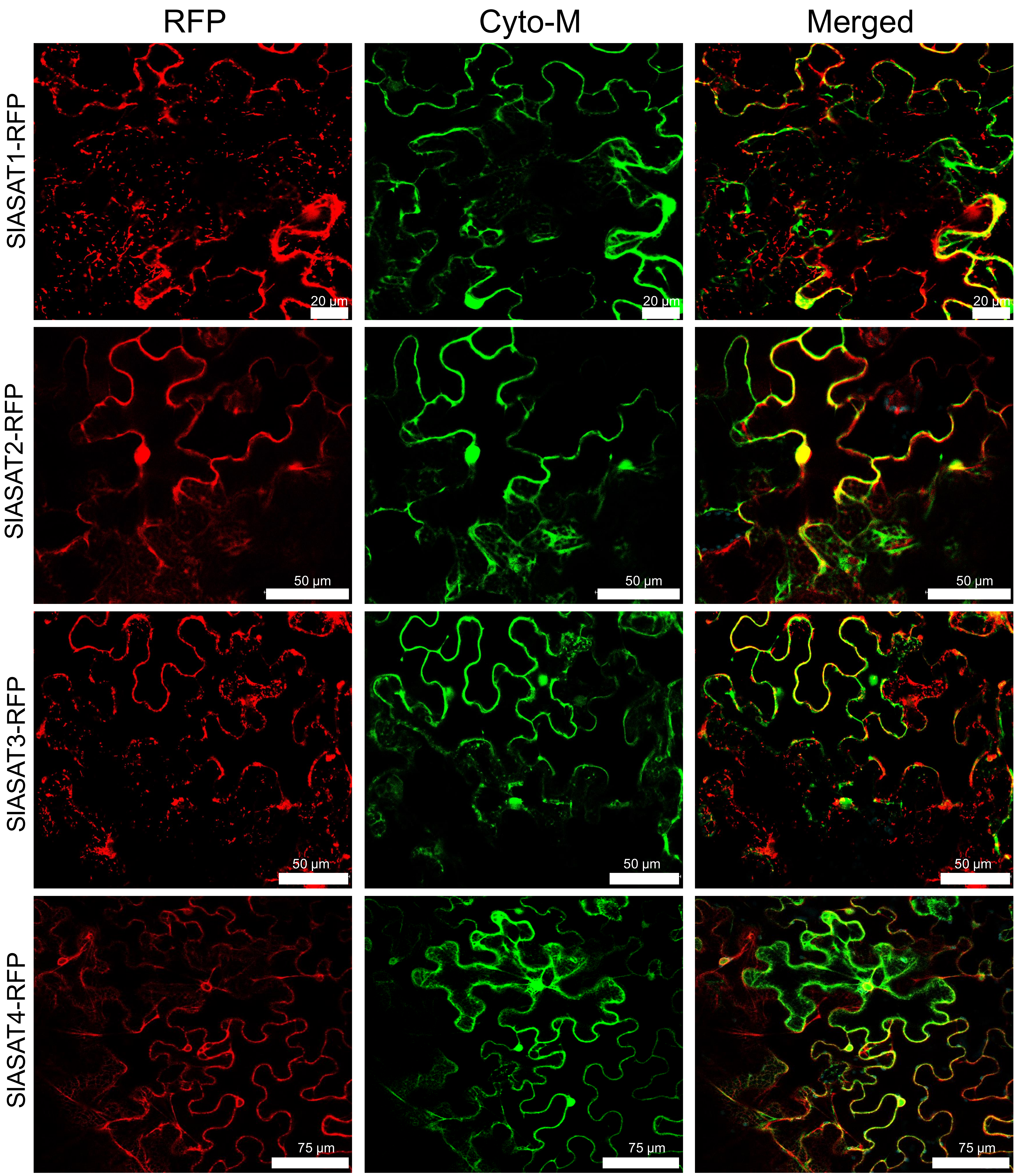

Fig. S4. Subcellular localization of SlASAT1-4-RFP-tagged proteins in *N. benthamiana* leaves with a cytosolic marker. SlASAT1-4-RFP fusion constructs were expressed in *N. benthamiana* leaves and the localization of the corresponding proteins were detected by confocal laser scanning microscopy 3 days after infiltration. The "RFP" panels (red) represent the signals of SlASAT1–4 fluorescent fusion proteins; the "Cyto-M" panels (green) represent the cytosolic marker signals; and the "Merged" panel shows the overlay of RFP and Cyto-M signals. Representative images are shown with a scale bar included in each image.

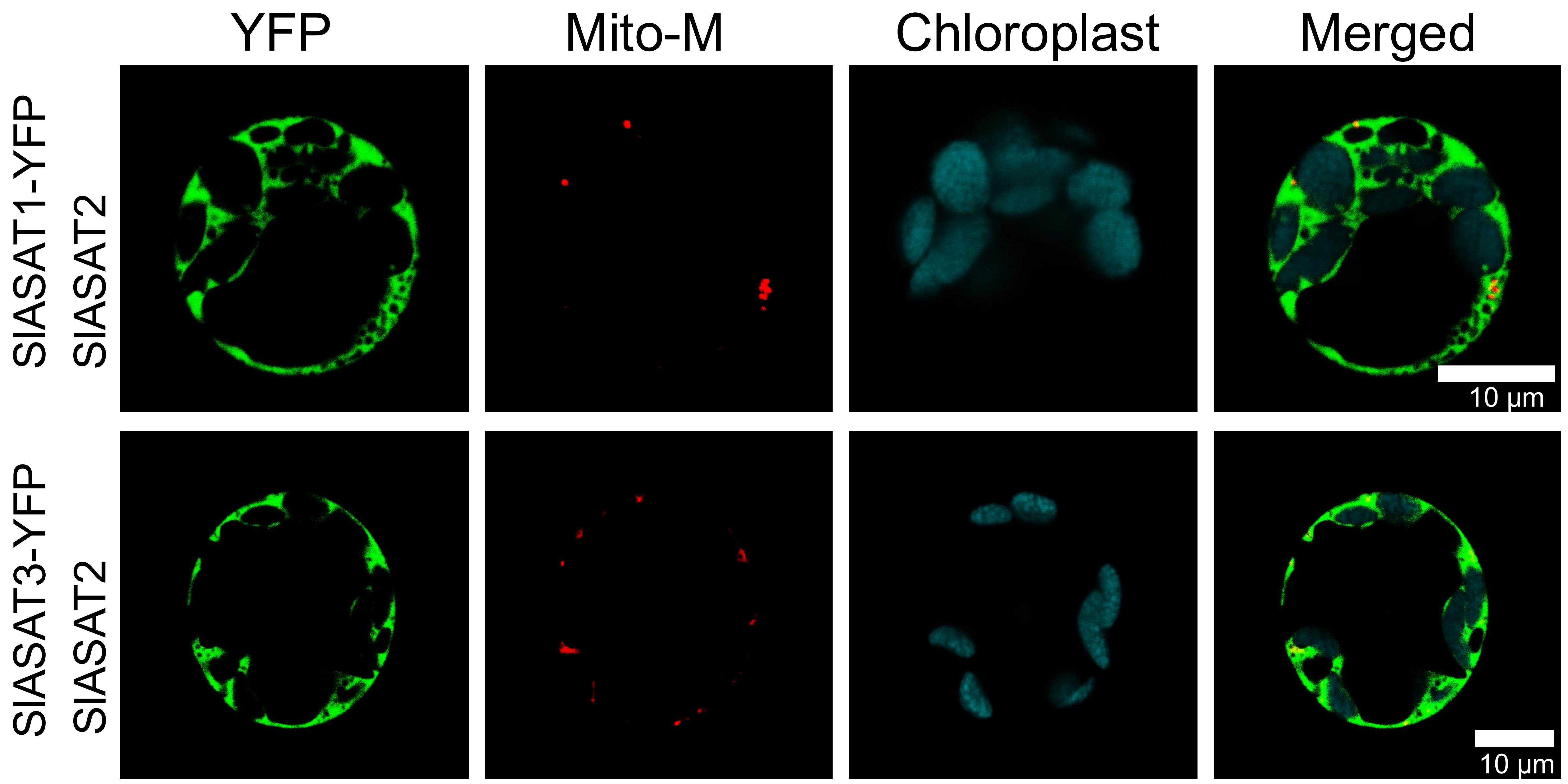

Fig. S5. SlASAT2 alters SlASAT1 and SlASAT3 subcellular localization to the cytosol. Co-expression of SlASAT1-YFP and SlASAT3-YFP together with untagged SlASAT2 changed their subcellular localization, preventing them from co-localizing with the mitochondrial marker. Green represent signals of SlASATs fused YFP proteins; the “Mito-M” panels (red) represent the signals of mitochondrial marker; the “chloroplast” panels (cyan) represent chlorophyll autofluorescence. The “Merged” panel shows the overlay of YFP, Mito-M, and chloroplast signals. Representative images are shown with a scale bar included in each panel.

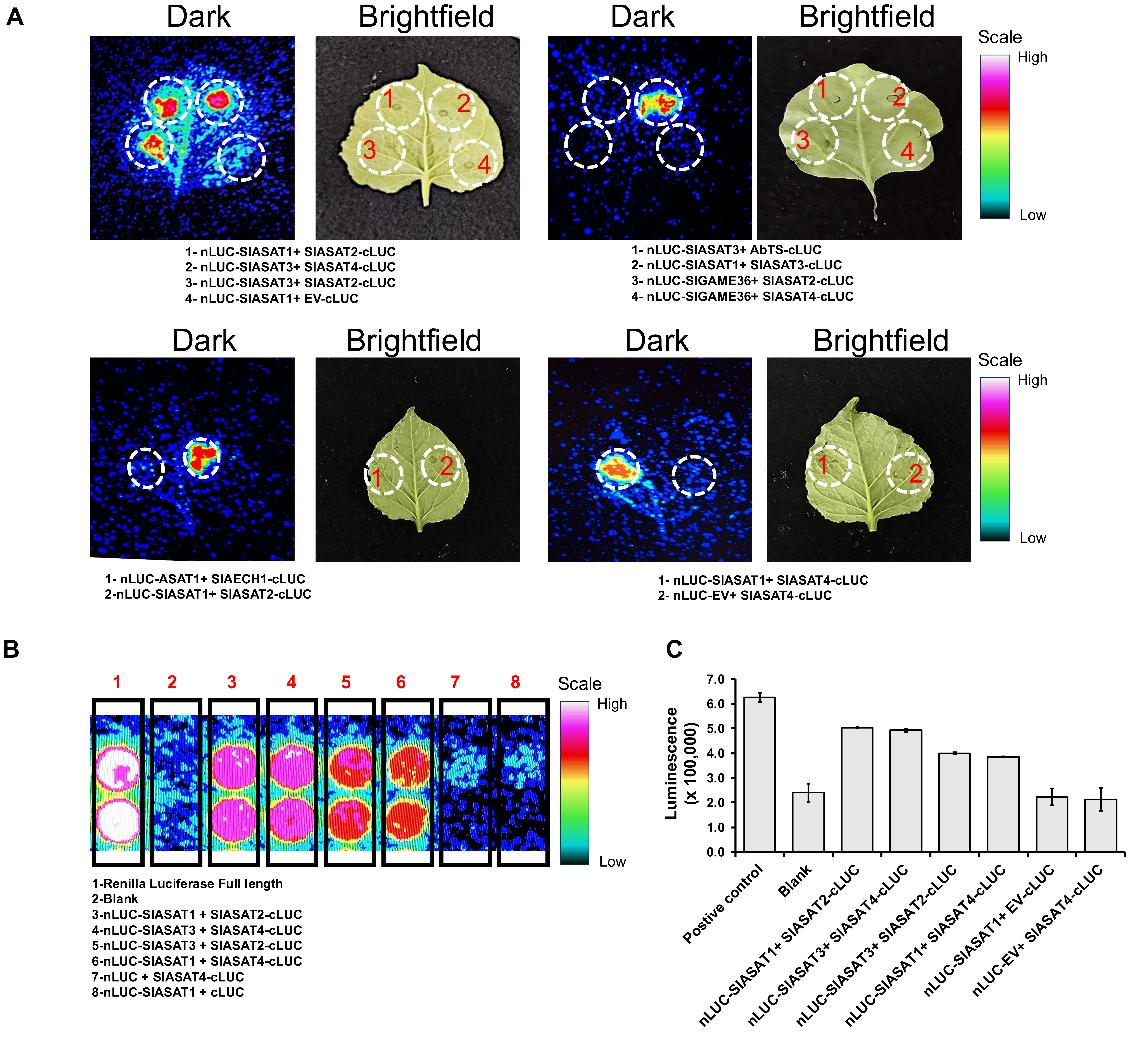

Fig. S6. SlASAT protein-protein interactions via split-luciferase assays in *N. benthamiana.* **A)** Split-luciferase assay images of *N. benthamiana* leaves co-infiltrated with *Agrobacterium* strains containing SlASATs following incubation for 3 days. Dotted circles indicate leaf panels that were infiltrated with *Agrobacterium* carrying the respective constructs. The luminescence intensity was recorded using a Photek-216. Combinations of SlASATs or negative controls are shown below each panel. **B)** Split-luciferase complementation assay in *N. benthamiana.* Split-luciferase assay images of protein extracts from *N. benthamiana* leaves expressing different SlASAT combinations. Proteins were extracted and placed in a 96 well plate and the luciferase activity was monitored in each well. The two replicate wells are shown for each construct. **C)** Quantification of the relative luminescence unit intensity for different combination of SlASATs using Image J. Bars indicate average ± standard error of 2 biological replicates. Scale bar indicates intensity of the observed luminescence.

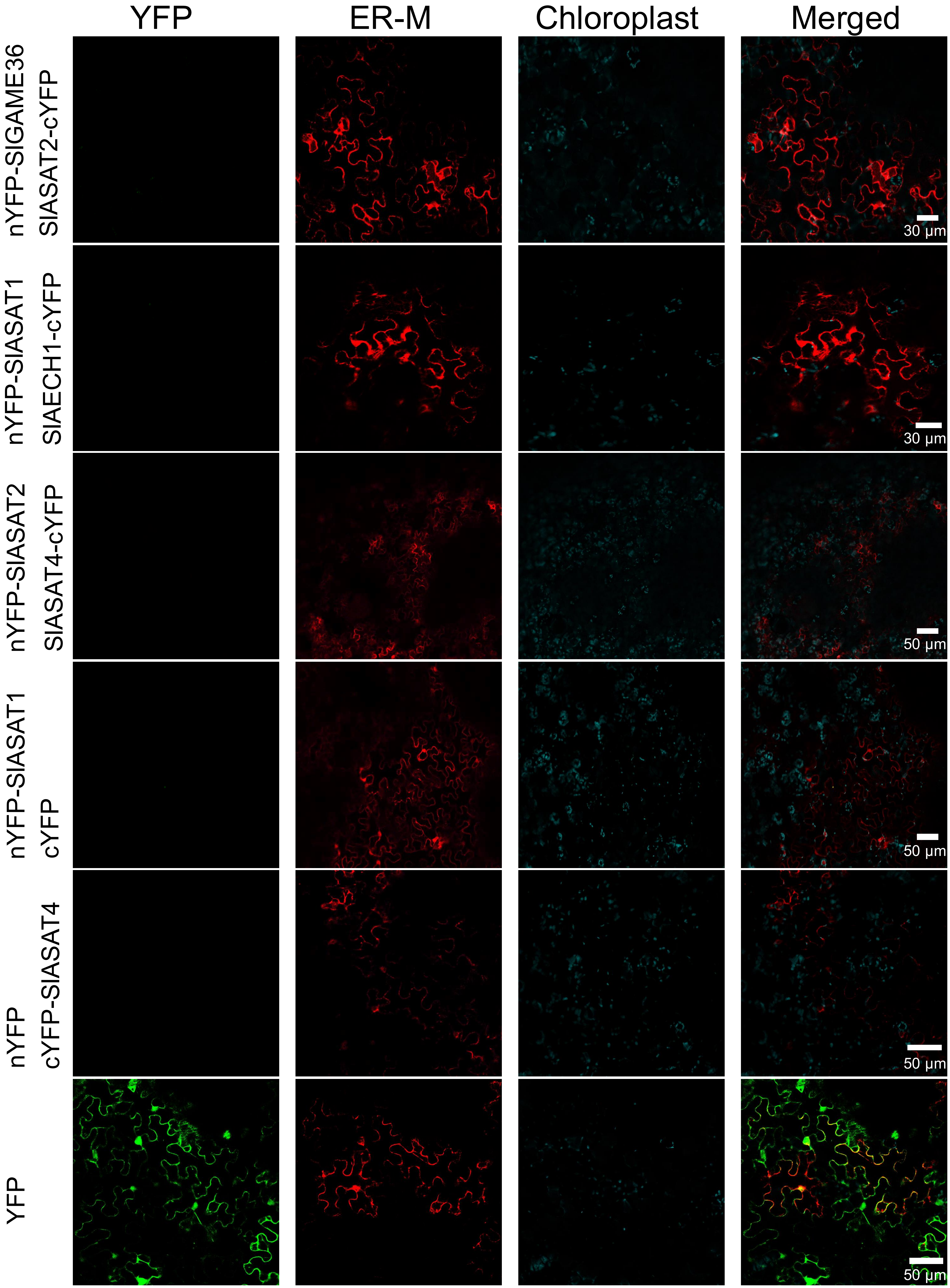

Fig. S7. SlASAT protein-protein interactions via BiFC in *N. benthamiana* with an ER marker. SlASAT1 and SlASAT3 were fused to N-terminal YFP, whereas SlASAT2 and SlASAT4 were fused to C-terminal YFP. Pairs of SlGAME36 + SlASAT2, SlAECH1 + SlASAT1, SlASAT1 + cYFP, and SlASAT4 + nYFP were used as negative controls. The “YFP” panels (green) represent reconstituted YFP fluorescence; the “ER-M” panels (red) show the ER marker signals (AtWAK2-RFP-HDEL); and the “chloroplast” panels (cyan) indicate chlorophyll autofluorescence. The “Merged” panel displays the overlay of YFP, ER-M, and chloroplast signals. Representative images are shown with a scale bar included in each panel.

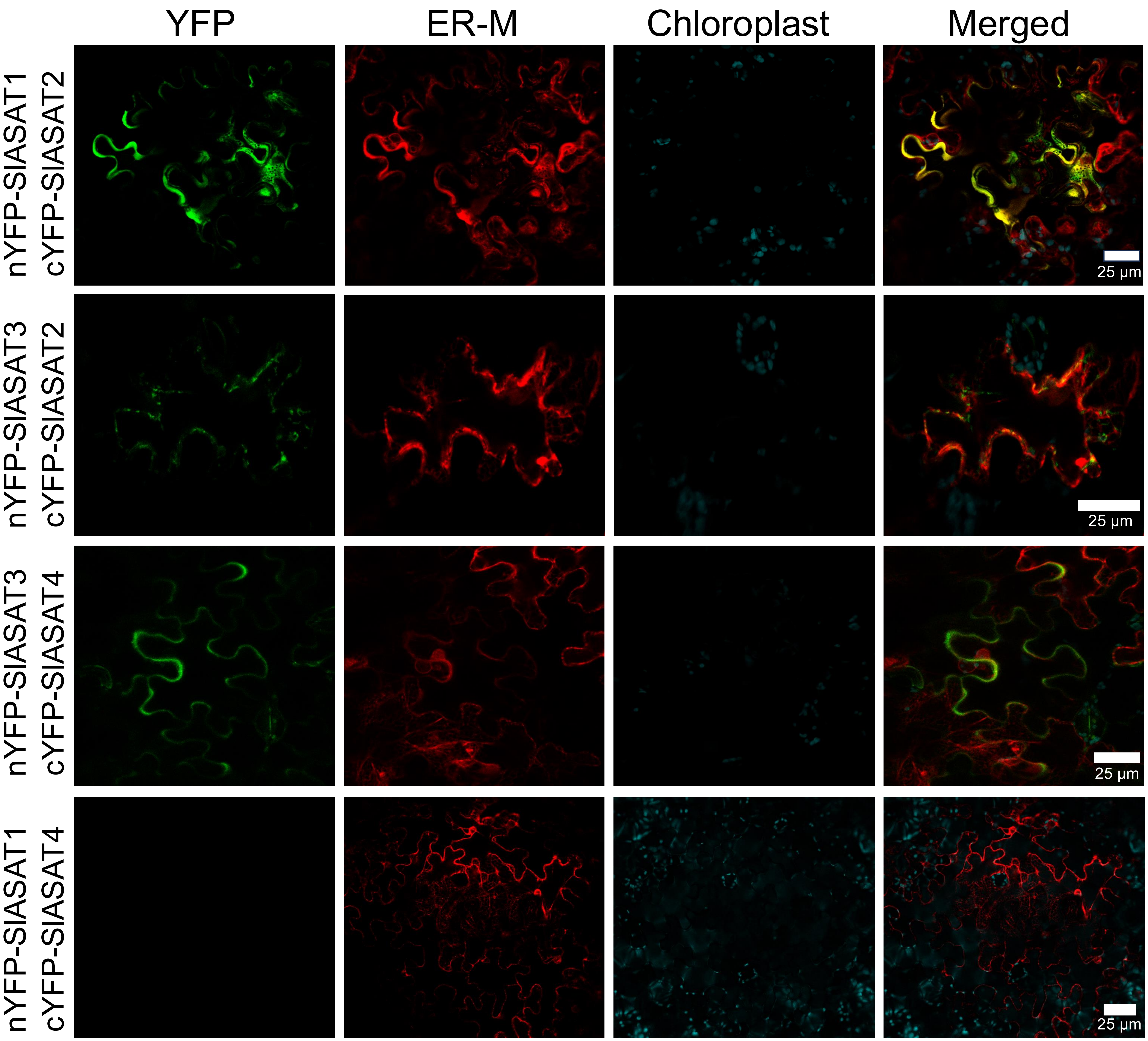

Fig. S8. Moving fluorescent tag locations demonstrates SlASAT pairwise interaction via BiFC assays in *N. benthamiana.* BiFC assays of the interaction between SlASATs transiently expressed in *N. benthamiana* leaves with an ER marker. SlASAT1 and SlASAT3 were fused to N-terminal YFP, while SlASAT2 and 4 were fused to C-terminal YFP. The "YFP" panels (green) represent signals of SlASATs complex; the "ER-M" panels (red) represent the signals of ER marker (AtWAK2- RFP-HDEL); the "chloroplast" panels (cyan) represent chlorophyll autofluorescence. The "Merged" panel displays the overlay of YFP, ER-M, and chloroplast signals. Scale bar shown in each panel of images. The experiments were repeated three times with similar results.

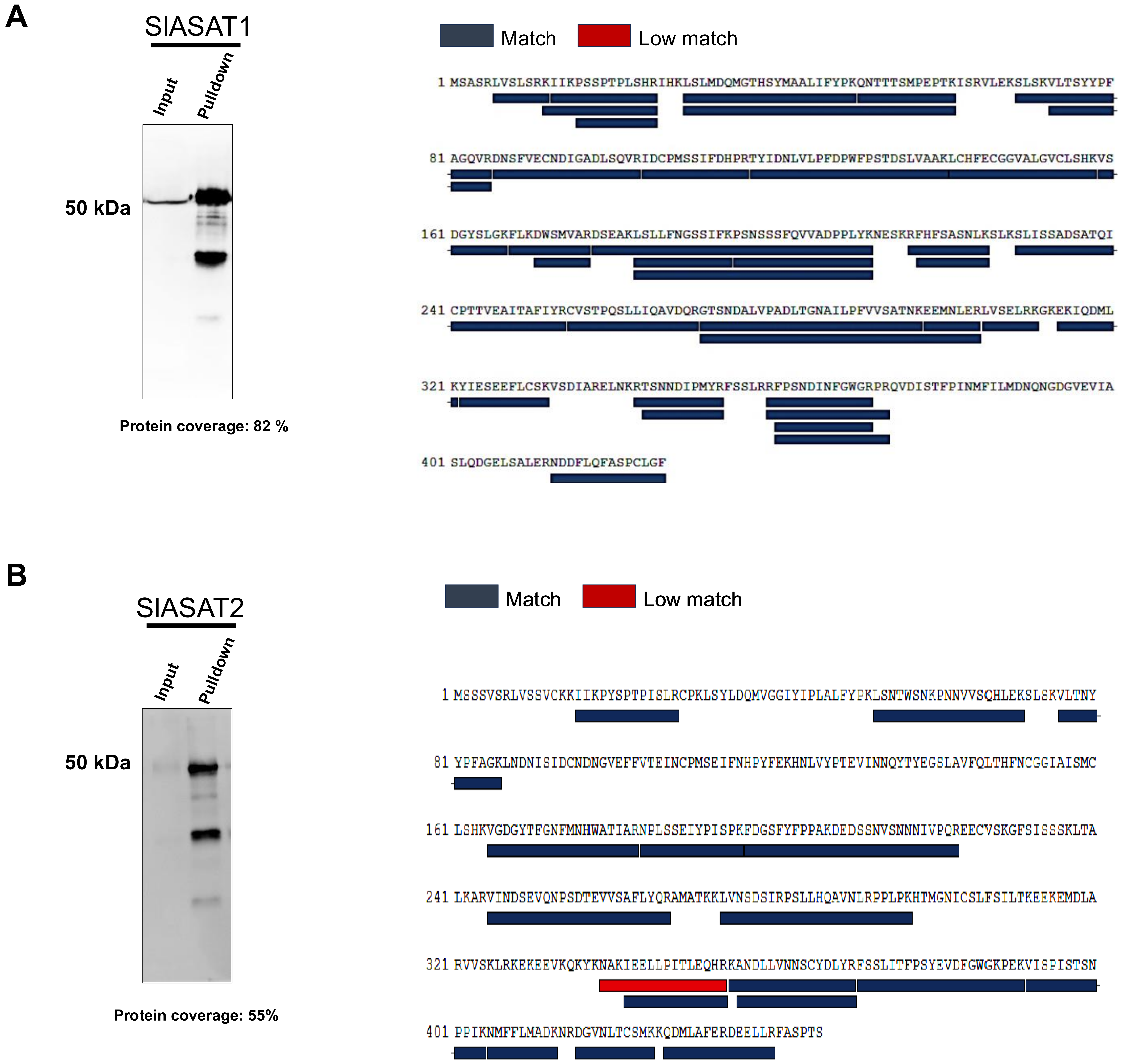

Fig. S9. SlASAT1 and SlASAT2 pull-down optimization. Protein gel blot and proteomics analysis of SlASAT1 and SlASAT2 pull-down. The protein gel blot analysis of the input and HA-SlASAT1 **A)** and FLAG-SlASAT2 **B)** immunoprecipitation (IP) samples, along with the schematic overview of the detected peptides corresponding to SlASATs using LC/MS-MS. IP was performed with anti-HA or anti-FLAG antibody. The experiment was repeated three times, with similar results. The peptides identified in the LC/MS-MS analysis are represented by dark blue boxes (high match > 90% amino acid identity), while the low match peptides are represented by red boxes (< 90% amino acid identity).

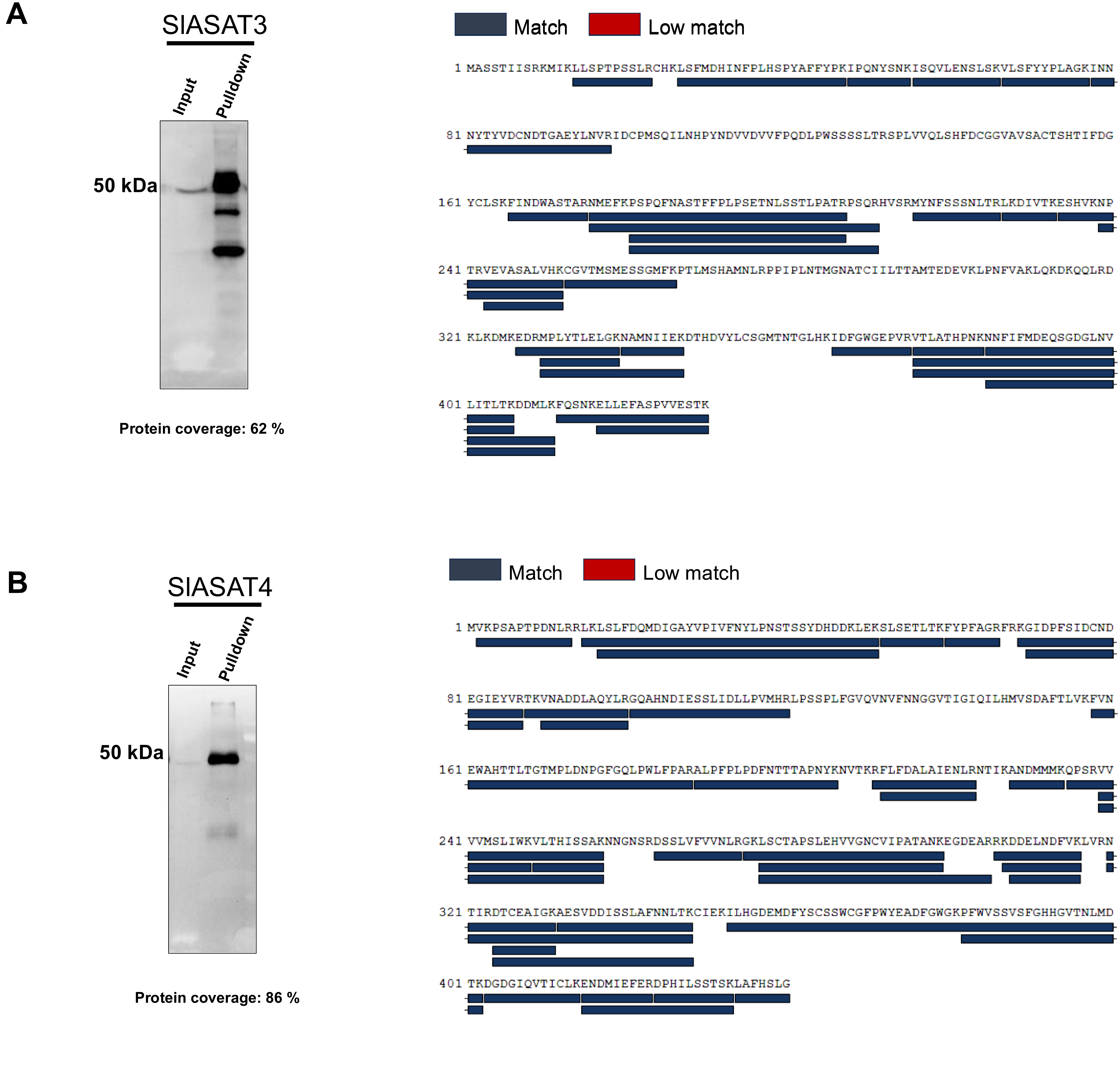

Fig. S10. SIASAT3 and SIASAT4 pull-down optimization. Protein gel blot and proteomics analysis of SIASAT3 and SIASAT4 pull-down. The protein gel blot analysis of the input and HA-SIASAT3 **A)** and FLAG-SIASAT4 **B)** immunoprecipitation (IP) samples, along with the schematic overview of the detected peptides corresponding to SIASAT proteins using LC/MS-MS. IP was performed with anti-HA or FLAG antibody. The experiment was repeated three times, with similar results. The peptides identified in the LC/MS-MS analysis are represented by dark blue boxes (high match > 90% amino acid identity), while the low match peptides are represented by red boxes (< 90% amino acid identity).

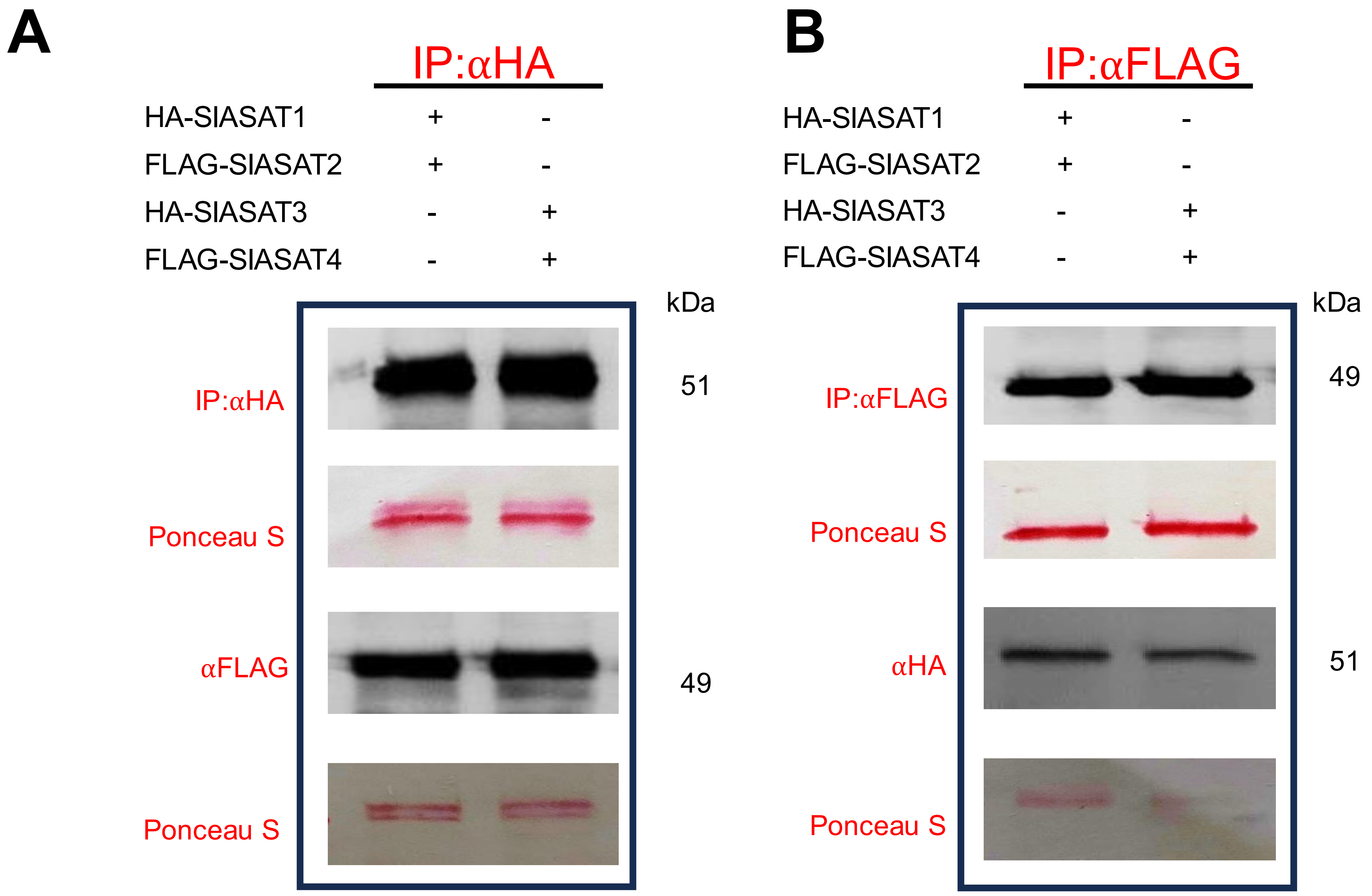

Fig. S11. Confirmation of pairwise SlASAT interactions via Co-IP. **A)** Co-immunoprecipitation of SlASATs show pairwise interactions. Protein extracts were transiently expressed in a pairwise manner: 35S::HA-SlASAT1 together with 35S::FLAG-SlASAT2, and 35S::HA-SlASAT3 together with 35S::FLAG-SlASAT4. Protein gel blot analysis of HA-IP samples. IP was performed with anti-HA magnetic bead conjugate and interacting proteins were analyzed with an anti-FLAG antibody. **B)** Protein gel blot analysis of FLAG-IP samples. IP was performed with anti-FLAG magnetic bead conjugate, and interacting proteins were analyzed with an anti-HA antibody. Abbreviations*:* IP, Immunoprecipitation; HA, Hemagglutinin. The experiment was repeated three times with similar results.

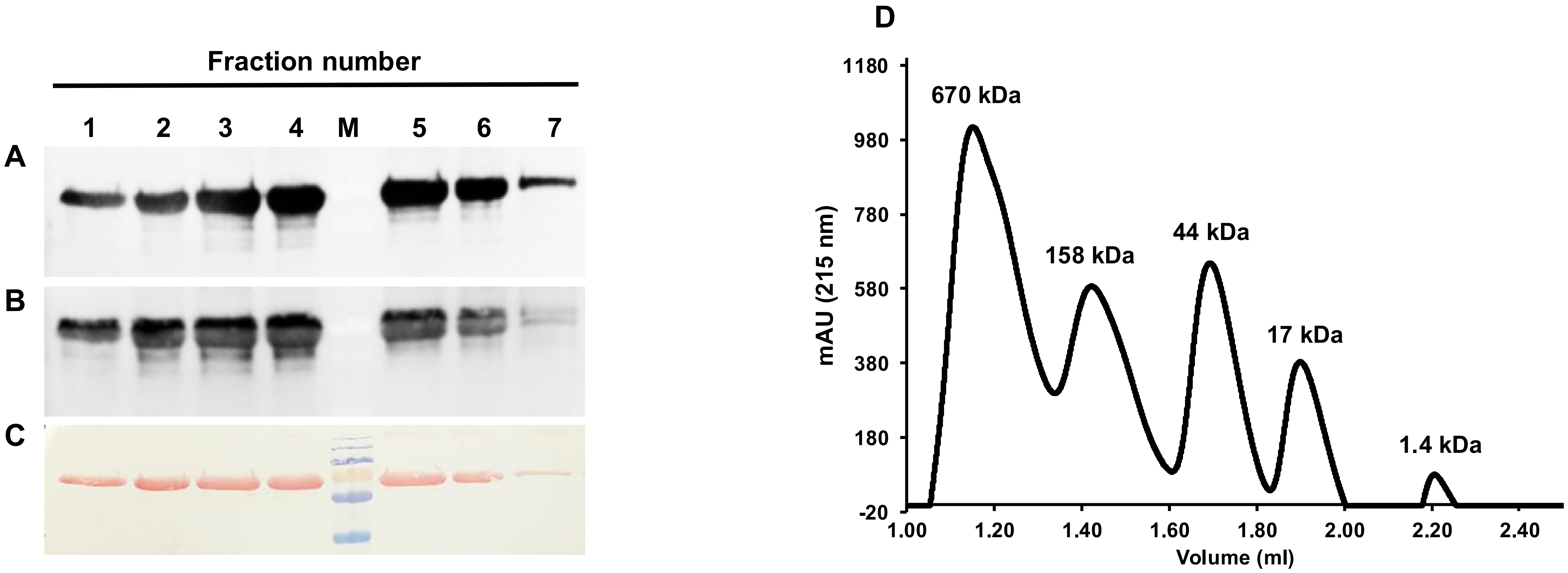

Fig. S12. Size exclusion chromatography of the SlASAT pulldown complex. Protein extracts were prepared from *N. benthamiana* leaves transiently expressing a combination of 35S::HA-SlASAT1, 35S::SlASAT2, 35S::SlASAT3, and 35S::FLAG-SlASAT4, the pull-down complex was eluted using non-denaturing conditions with HA peptide and separated over size exclusion chromatography (SEC). **A)** Protein gel blot analysis using anti-HA antibody of various SEC fractions showing the abundance of SlASAT1. **B)** Protein gel blot analysis using anti-FLAG antibody of various SEC fractions showing the abundance of SlASAT4. **C)** The gel blot was stained with Ponceau S. **D)** A set of globular protein standards was separated using the identical size exclusion column and method as used in Fig. 5B for the separation of the SlASAT1-4 complex. The molecular masses of the protein standards are indicated in kDa above each peak. The protein standards were monitored using a 215 nm wavelength.

Protein ID	Protein Descriptions
A0A060IKL	fatty acid biosynthetic process
NbPDR2a	ABC-type transporter activity
NbG6PDH	glucose-6-phosphate dehydrogenase activity
E5LLE7	Phosphoglycerate kinase
NbMVD1a	isoprenoid biosynthetic process
NbACAT1a	acetyl-CoA C-acetyltransferase activity
NbPDR2b	ABC-type transporter activity
A0A1U9IKY9	mitochondria-associated ER membrane contact site
A0A288UJC0	acyltransferase activity
NbRanBP1-1a	nucleocytoplasmic transport

Fig. S13. List of candidate proteins identified through proteomics analysis of the SlASAT1-4 metabolic complex. These candidates were common across all replicates and exhibited high abundances. The full list of identified proteins is provided in Supplementary Table 2.
